# Differential role of SIRT1/MAPK pathway during cerebral ischemia in rats and humans

**DOI:** 10.1038/s41598-021-85577-9

**Published:** 2021-03-18

**Authors:** Sireesh Kumar Teertam, Phanithi Prakash Babu

**Affiliations:** Department of Biotechnology and Bioinformatics, School of Life Sciences, University of Hyderabad, Prof. C. R. Rao Road, Gachibowli, Hyderabad, TS 500 046 India

**Keywords:** Stroke, Stroke

## Abstract

Cerebral ischemia (CI) is a severe cause of neurological dysfunction and mortality. Sirtuin-1 (Silent information regulator family protein 1, SIRT1), an oxidized nicotinamide adenine dinucleotide (NAD^+^)-dependent protein deacetylase, plays an important role in protection against several neurodegenerative disorders. The present study aims to investigate the protective role of SIRT1 after CI in experimental young and aged rats and humans. Also, the study examines the possible regulatory mechanisms of neuronal death in CI settings. Immunoblotting and immunohistochemistry were used to evaluate changes in the expression of SIRT1, JNK/ERK/MAPK/AKT signaling, and pro-apoptotic caspase-3 in experimental rats and CI patients. The study findings demonstrated that, in aged experimental rats, SIRT1 activation positively influenced JNK and ERK phosphorylation and modulated neuronal survival in AKT-dependent manner. Further, the protection conferred by SIRT1 was effectively reversed by JNK inhibition and increased pro-apoptotic caspase-3 expression. In young experimental rats, SIRT1 activation decreased the phosphorylation of stress-induced JNK, ERK, caspase-3, and increased the phosphorylation of AKT after CI. Inhibition of SIRT1 reversed the protective effect of resveratrol. More importantly, in human patients, SIRT1 expression, phosphorylation of JNK/ERK/MAPK/AKT signaling and caspase-3 were up-regulated. In conclusion, SIRT1 could possibly be involved in the modulation of JNK/ERK/MAPK/AKT signaling pathway in experimental rats and humans after CI.

## Introduction

Ischemic stroke (IS) or cerebral ischemia (CI) is the most common subtype of stroke^1^ and can affect individuals of any age. It is well observed that older individuals have the highest incidence of IS and also poor functional recovery^2^. Majority of the stroke studies are conducted in young experimental animal models. Various neuroprotective compounds have been shown to be effective in young experimental models in preclinical studies; however, these compounds failed in clinical trials. The molecular mechanisms of injury and risk factors for stroke vary in rodents and humans^3^. A combined assessment of experimental procedures in both models could conceivably avoid ambiguity and provide a better understanding of stroke pathophysiology.

Sirtuins are evolutionarily conserved class-III oxidized nicotinamide adenine dinucleotide (NAD^+^)-dependent histone and nonhistone deacetylases. There are seven homologs (SIRT1–SIRT7) with different enzymatic activities and distribution in mammalian cells. Silent information regulator family protein 1 (SIRT1) is the best-characterized sirtuin among the seven homologs and predominantly expressed in neurons and adult brain^4,5^. SIRT1 plays a significant role in longevity and cell survival by deacetylation of various stress-responsive transcription factors related to inflammation and apoptosis^6,7^. Modulation of SIRT1 activity by genetic and pharmacological means has been shown to be protective in neurodegenerative disease models. The brain-specific knockdown of SIRT1 has been reported to exacerbates Huntingtin disease pathology in an R6/2 mouse model via BDNF/CREB signaling^8^. Resveratrol, a SIRT1 activator, reportedly protects the amyotrophic lateral sclerosis (ALS) cell model from mutant superoxide dismutase 1 (SOD1)-mediated toxicity by up-regulation of SIRT1 expression^9^.

Recent studies have found that mitogen-activated protein kinase (MAPK) signaling plays a critical role in the progression of CI^10,11^. CI activates the c-JUN N-terminal kinase (JNK), a subgroup of the MAPK family, which contributes to the apoptotic cell death^12^. On the other hand, several growth factors are associated with the activation of signal transduction pathways such as extracellular signal regulated kinase (ERK), a subfamily of MAPK family, and IRS/AKT pathway^13^. ERK is responsive to growth stimuli and contributes to neuronal cell proliferation and differentiation^14^. It also acts as a key regulator of apoptotic neuronal death^15^. Under the influence of growth factors, IRS-1 activates PI3K and subsequently recruits AKT, and AKT activation decreases neuronal cell death after CI and, thereby, negatively regulates MEK/ERK/MAPK pathway^16,17^. Previous studies have shown that SIRT1 directly affects AKT activity and promotes neuronal differentiation^18^. In recent times, the possible correlation between SIRT1 activation with MAPK signaling has been investigated in both cardiomyocytes and hepatic ischemia^19,20^. It has been shown that icariin enhances neuronal survival after oxygen–glucose deprivation by the activation of p38/MAPK/SIRT1 signaling^21^. SIRT1 inhibition has been shown to reduced IGF-I/IRS-2/Ras/ERK signaling and protects neurons from oxidative stress^22^, suggesting the possible involvement of SIRT1 and MAPK signaling in neuronal survival. Thus, the present study investigates the possible involvement of both SIRT1 and JNK/ERK/MAPK/AKT signaling in neuronal apoptosis in human patients and ischemic brains of rats subjected to middle cerebral artery occlusion (MCAO).

## Results

### Decrease in SIRT1 expression and phosphorylation of JNK/ERK/MAP kinases in brains of experimental aged rat following MCAO

**Figure 1:**
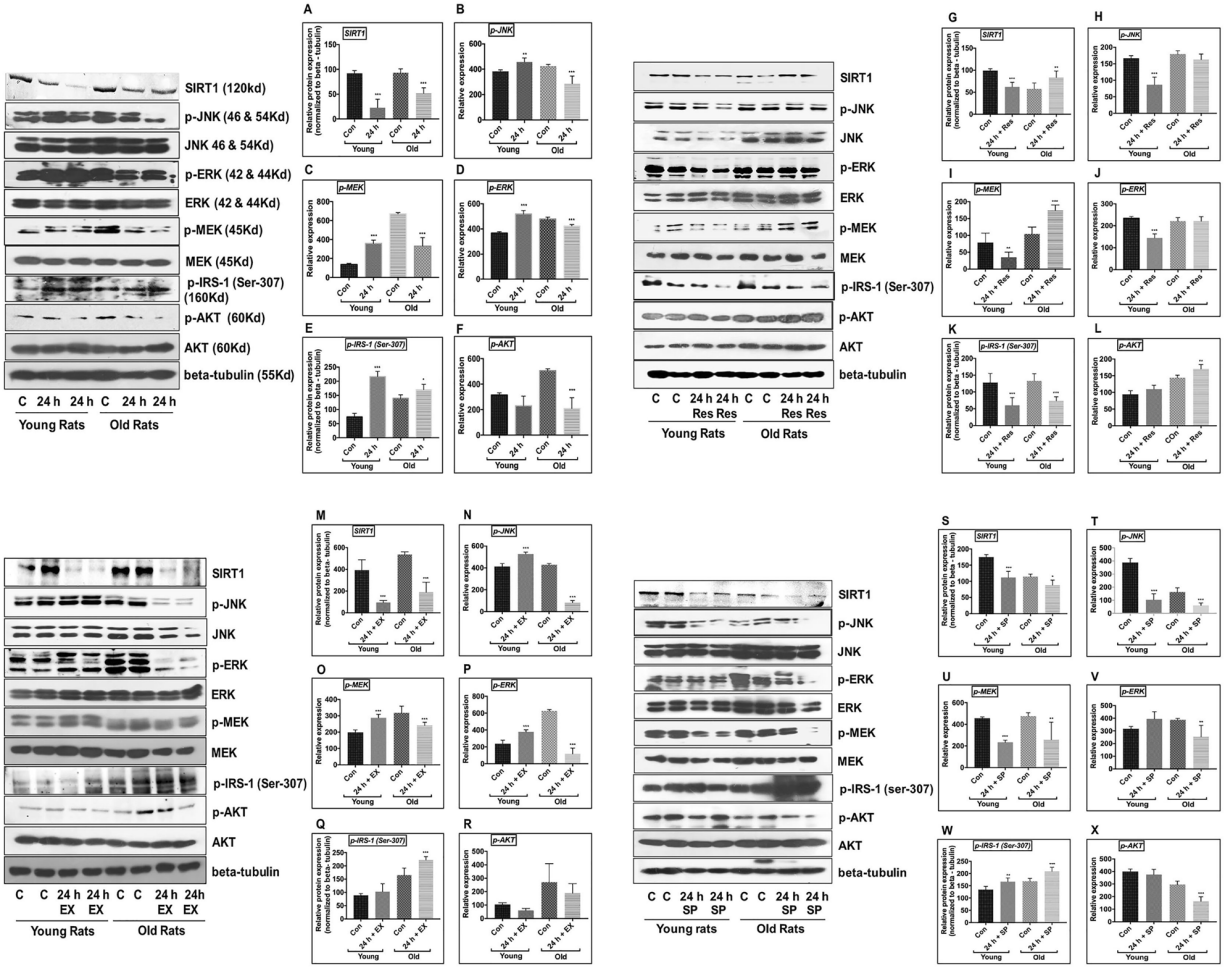
Effect of ischemia on the expression of SIRT1, JNK/ERK/MAPK/AKT signaling pathway in brains of control, 24 h MCAO, MCAO + EX-527, MCAO + resveratrol, and MCAO + SP600125 treatment in young and aged rat. Respective total proteins and beta-tubulin were served as the loading control (p-JNK, p-MEK, p-ERK, and p-AKT), while SIRT1 and p-IRS-1 were normalized to beta-tubulin. **(A–F)** Statistical results on the changes in expression of SIRT1, p-JNK, p-MEK, p-ERK, p-IRS-1, and p-AKT expression following CI. **(G–L)** Statistical results on the expression of SIRT1, p-JNK, p-MEK, p-ERK, p-IRS-1, and p-AKT on resveratrol treatment. **(M–R)** statistical results on the expression of SIRT1, p-JNK, p-MEK, p-ERK, p-IRS-1, and p-AKT on SIRT1 inhibition with EX-527. **(S–X)** Statistical results on the expression of SIRT1, p-JNK, p-MEK, p-ERK, p-IRS-1, and p-AKT on JNK inhibition with SP600125 treatment. The densitometry values are represented as mean ± SD (n = 5). *p < 0.05 versus sham control.

SIRT1 protein expression, assessed using western blot analysis, was significantly decreased in the ischemic brains of young and aged experimental rats compared with sham control (SIRT1-p < 0.001, Fig. 1A). To explore the involvement of MAPKs in the pathogenesis of ischemia, the phosphorylation status of MAPKs, including JNK, MEK, and ERK was examined using western blotting. In aged rats following MCAO, JNK/ERK/MAPK phosphorylation was substantially decreased compared with sham control (p-JNK-p < 0.001; p-MEK- p < 0.001; p-ERK-p < 0.02, Fig. 1B–D).

On the contrary, young ischemic rats exhibited a significant increase in the phosphorylation status of stress-induced JNK/ERK/MAPK compared with sham controls (p-JNK-p < 0.007; p-MEK-p < 0.001; p-ERK-p < 0.001, Fig. 1B–D).

### Subcellular localization of SIRT1, p-JNK, and caspase-3 following MCAO in aged rats

**Figure 2:**
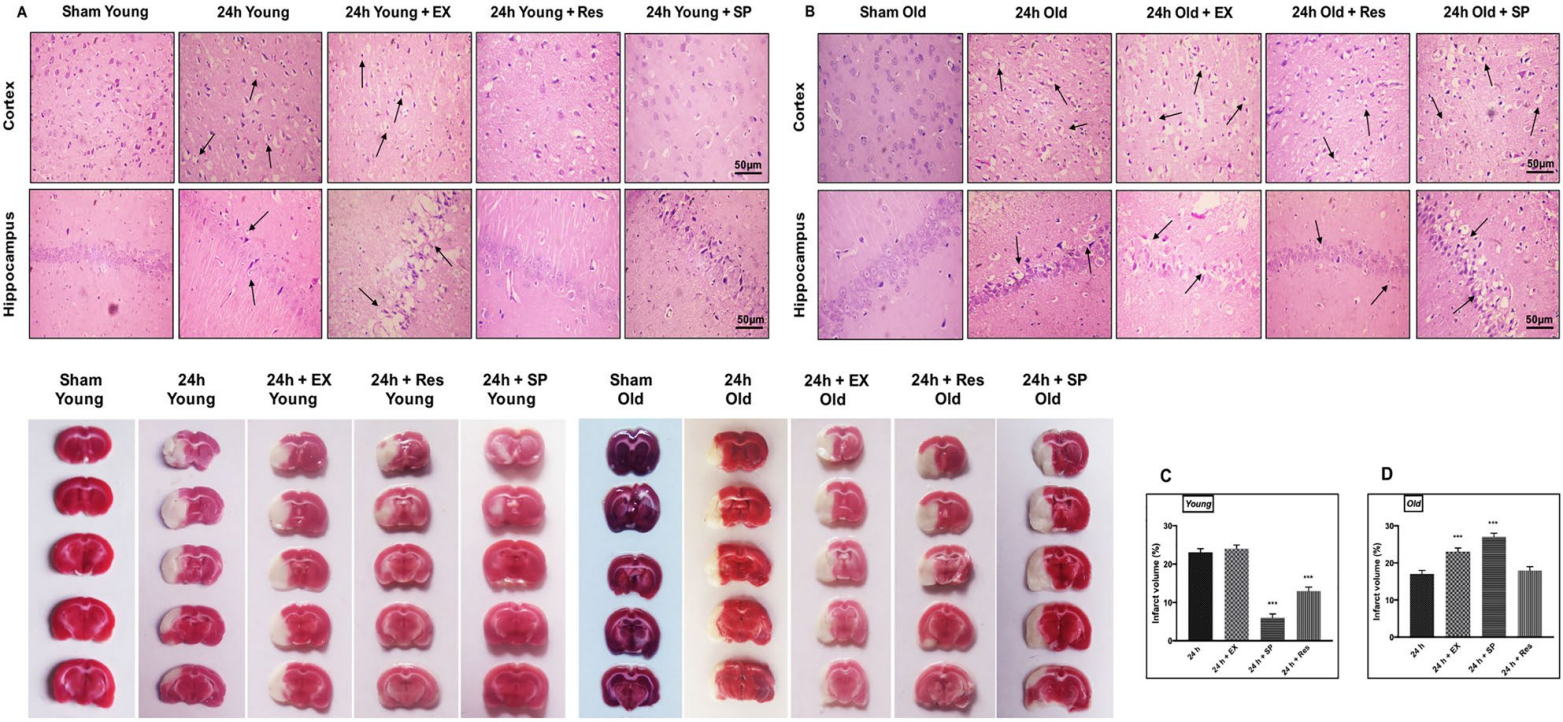
Assessment of morphological changes following CI. The morphological changes in cortex and hippocampus neurons were assessed using H&E staining (magnification 400X; Scale Bar—50 μm). **(A) **Representative H&E staining of paraffin-embedded brain sections from sham control, 24 h MCAO, MCAO + resveratrol, MCAO + EX-527, and MCAO + SP600125 treatment in young rats. Morphological changes were represented in black arrows (pyknotic neuron). Black arrow shows the presence of extensive changes in neuronal morphology in the brains of 24 h MCAO and EX-527 treatment, whereas pathological changes was decreased in resveratrol and Sp600125 treatment. **(B)** H&E staining of brain sections from sham control, 24 h MCAO, MCAO + resveratrol, MCAO + EX-527, and MCAO + SP600125 treatment in aged rats. Black arrow represents the presence of extensive changes in neuronal morphology in the brains of 24 h MCAO and EX-527 treatment, whereas SP600125 and resveratrol failed to reduce pathological changes. The infarct intensity was measured with TTC staining. **(C)** Resveratrol and SP600125 treatment decreased infarct volume and abolished the effect of EX-527 in the brains of young rat following MCAO. **(D)** In aged experimental rats EX-527 and SP600125 treatment increased infarct and resveratrol treatment failed to reduce infarct following MCAO. The data are presented as a percentage of tissue loss (n = 3). *p < 0.05 versus ischemic brain.

**Figure 3:**
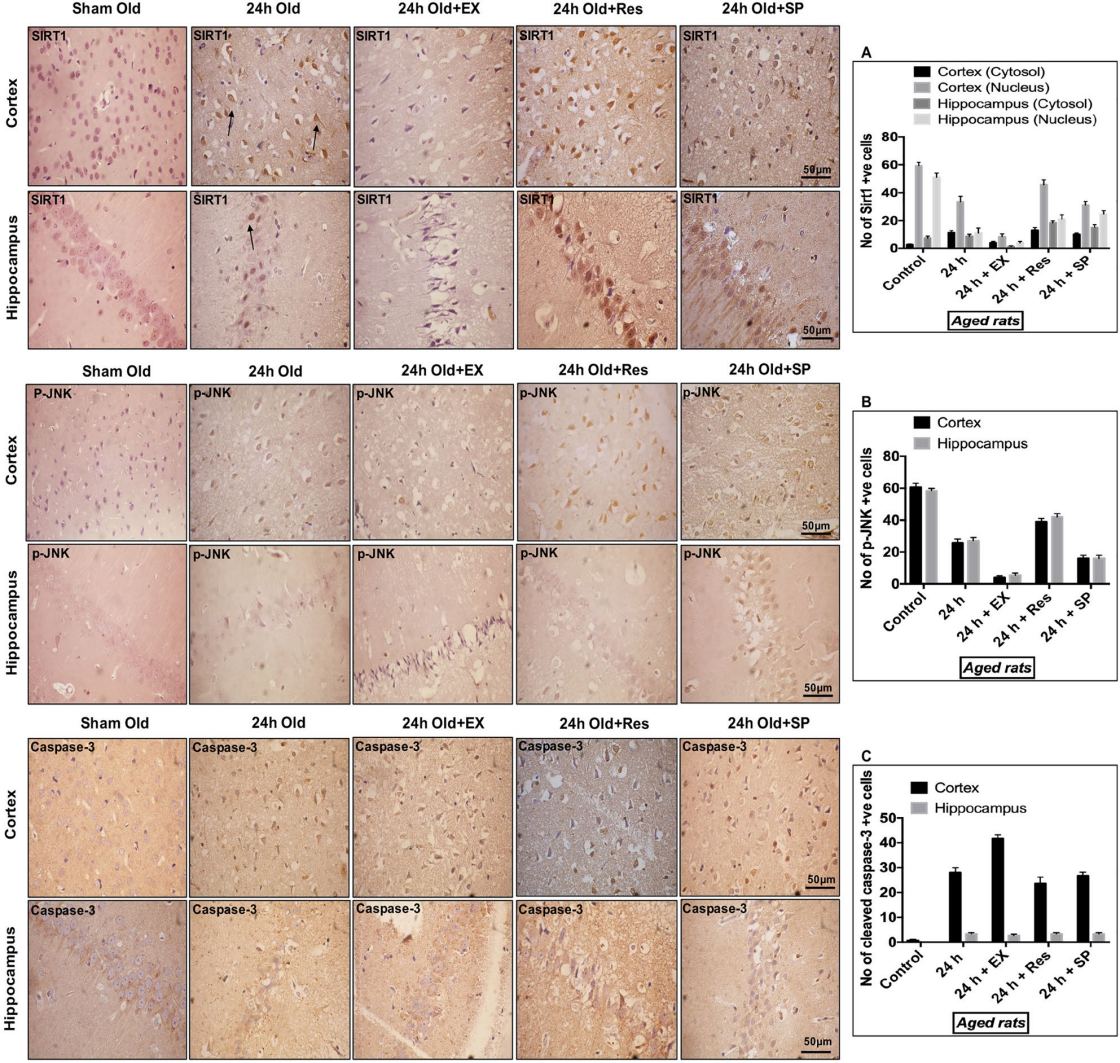
Subcellular localization of SIRT1, p-JNK, & caspase-3 in aged rats following CI. Aged rat brains were paraffin embedded and sectioned into 5–10 μm thick slices (n = 4). **(A)** Subcellular distribution of SIRT1 in the brains of aged rat cortex and hippocampus neurons from sham control as well as brains from 24 h after MCAO, MCAO + EX-527, MCAO + resveratrol, and MCAO + SP600125 treatment. **(B)** p-JNK immunoreactivity in the brains of aged rat cortex and hippocampus neurons from control as well as brains from 24 h after MCAO, MCAO + EX-527, MCAO + resveratrol, and MCAO + SP600125 treatment. **(C)** Nuclear immunoreactivity of activated caspase-3 in the brains of aged rat cortex and hippocampus neurons from control as well as brains from 24 h after MCAO, MCAO + EX-527, MCAO + resveratrol, and MCAO + SP600125 treatment (magnification 400X; Scale Bar—50 μm). Immunoreactivity for SIRT1, p-JNK, and caspase-3 was depicted in brown (DAB). The neuronal nucleus is visualized with Hematoxylin counterstain.

**Figure 4:**
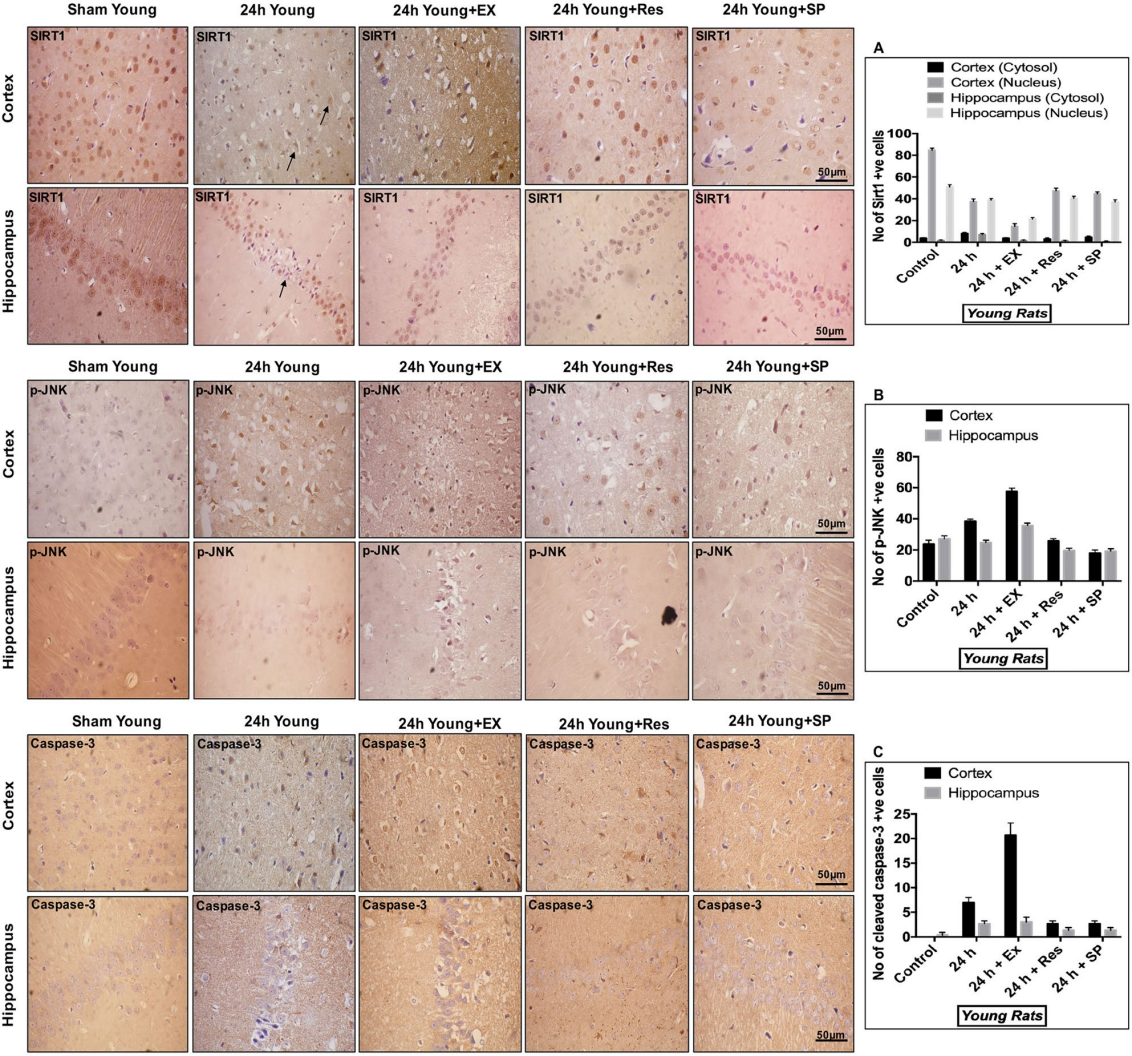
Subcellular localization of SIRT1, p-JNK, & caspase-3 in young rats following CI. Young rat brains were paraffin embedded and sectioned into 5–10 μm thick slices (n = 4). **(A)** Subcellular distribution of SIRT1 in the brains of young rat cortex and hippocampus neurons from sham control as well as brains from 24 h after MCAO, MCAO + EX-527, MCAO + resveratrol, and MCAO + SP600125 treatment. **(B)** p-JNK immunoreactivity in the brains of young rat cortex and hippocampus neurons from control as well as brains from 24 h after MCAO, MCAO + EX-527, MCAO + resveratrol, and MCAO + SP600125 treatment. **(C)** Nuclear immunoreactivity of activated caspase-3 in the brains of young rat cortex and hippocampus neurons from control as well as brains from 24 h after MCAO, MCAO + EX-527, MCAO + resveratrol, and MCAO + SP600125 treatment (magnification 400X; Scale Bar—50 μm). Immunoreactivity for SIRT1, p-JNK, and caspase-3 was depicted in brown (DAB). The neuronal nucleus is visualized with Hematoxylin counterstain.

To determine the cellular distribution of SIRT1, p-JNK, and caspase-3 in rats following ischemia, we immunostained paraffin-embedded brain sections of sham controls and MCAO (24 h) rats from both the age groups (young and aged rats). Routine hematoxylin and eosin (H&E) staining showed changes in neuronal morphology in both cortex and hippocampus following MCAO (Fig. 2A,B). The infarct size was assessed using 2,3,5-triphenyltetrazolium chloride (TTC) staining. A significant amount of cerebral infarction was observed following MCAO (Fig. 2C,D). Immunohistochemistry was performed against SIRT1, p-JNK, and caspase-3. SIRT1 immunoreactivity was predominantly observed in neuronal nuclei of cortex and hippocampus neurons in controls. The post-ischemic nuclear immunoreactivity was decreased in infarcted brains, and positive immunoreactivity was also observed in cytoplasm and cell processes of neurons in both young and aged experimental rats (Figs. 3A, 4A).

With respect to JNK in aged rats, the immunoreactivity of p-JNK was decreased following MCAO. On the contrary, in young experimental rats, the immunoreactivity of p-JNK was increased (Figs. 3B, 4B). The immunoreactivity for activated caspase-3 with a condensed nucleus and contracted cytoplasm is indicative of apoptotic cell death. A few caspase-3 positive cells were observed from neuronal cytoplasm of controls, and caspase-3 immunoreactivity was increased in infarcted brains. Furthermore, immunoreactivity was observed in cytosol and nucleus in both the age groups (Figs. 3C, 4C).

### Increase in SIRT1 expression and JNK/ERK/MAPK/AKT phosphorylation in post-mortem human stroke brain tissue

**Figure 5:**
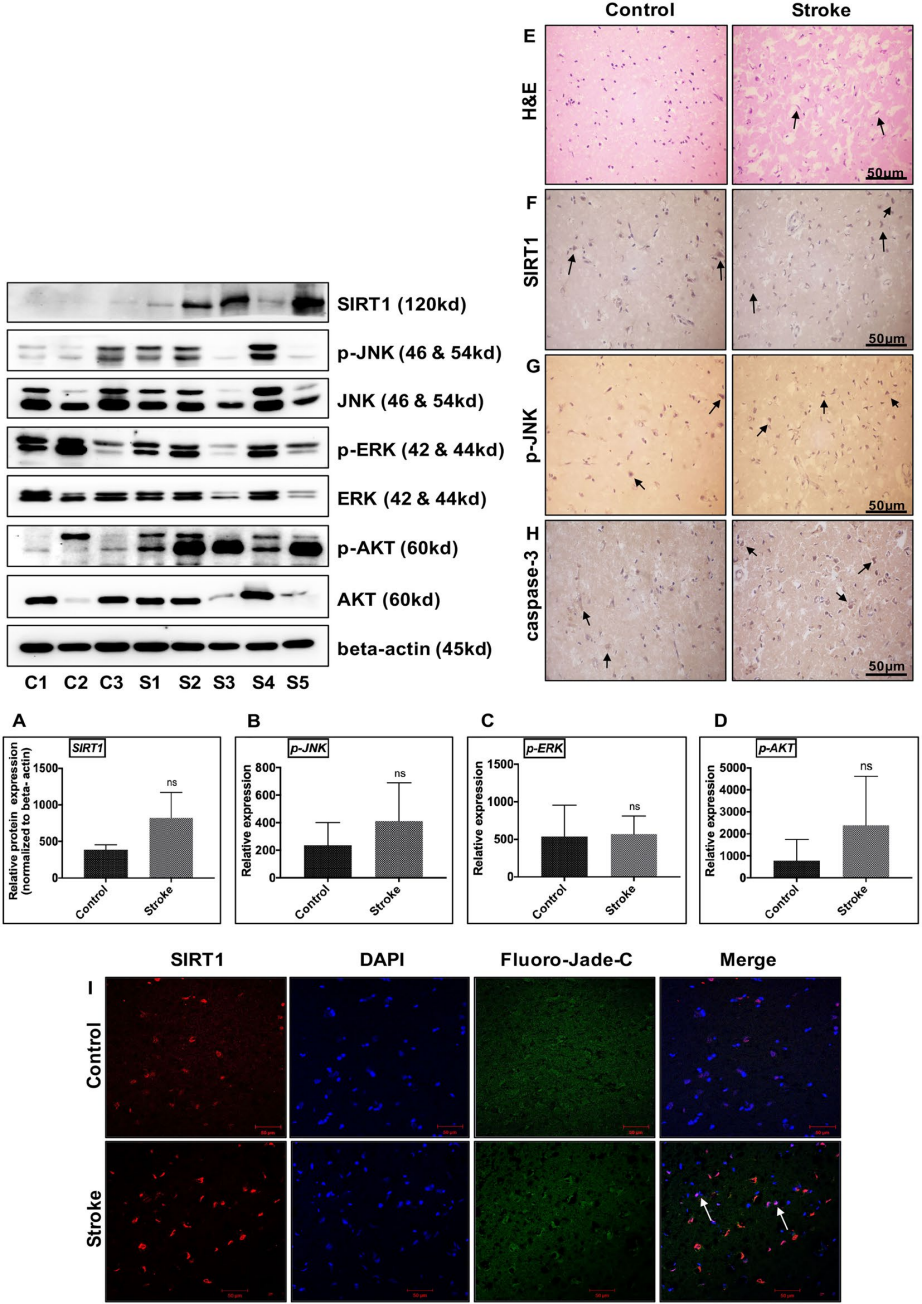
Effect of ischemia on the expression of SIRT1/JNK/ERK/AKT signaling pathway in Post-mortem human brain tissue. An equal amount of total protein sample (50 μg) from Control (n = 3) and Stroke brain (n = 5) were probed by western blots to analyze the expression of SIRT1 and phosphorylation status of p-JNK, p-ERK, and p-AKT. Respective total proteins and beta-actin were served as the loading control (p-JNK, p-ERK, and p-AKT), while SIRT1 was normalized to beta-actin. **(A–D)** represents an increase in SIRT1, p-JNK, p-ERK, and p-AKT protein expression in human stroke brain compared to respective healthy control. Data represented as mean ± SD. *p < 0.05 versus control brain. Human control (n = 3), as well as stroke brains (n = 4), were paraffin embedded and sectioned into 5-10 μm thick slices. **(E)** Histopathological changes were observed using H&E staining (magnification ×400; Scale Bar—50 μm). **(F,G,H)** immunoreactivity of SIRT1, p-JNK, and caspase-3 in control and stroke human brain. Black arrow represents changes in immunoreactivity from respective group. Hematoxylin counterstain used for visualization of neuron nucleus. Immunoreactivity for SIRT1, p-JNK, and caspase-3 was depicted in brown (DAB) (magnification ×400; Scale Bar—50 μm). **(I)** Triple immunofluorescence labeling was used to probe for SIRT1 (Red), DAPI (Blue), and Fluoro Jade-C (Green) in control (n = 3) and stroke human brains (n = 4). The representative image has shown the number of Fluoro Jade-C positive cells that co-localized with SIRT1 in stroke brain compared to control (white arrows), (Scale Bar—50 μm).

To explore the involvement of SIRT1, JNK/ERK/MAPK, and AKT in the pathogenesis of ischemia, the expression of SIRT1, phosphorylation status of JNK/ERK/MAPK, and AKT was examined using immunoblotting. We found that SIRT1 protein expression and phosphorylation of JNK/ERK/MAPK and AKT were upregulated in the stroke patients compared with the control (Fig. 5A–D).

The changes in neuronal morphology in stroke brain was showed using H&E staining of paraffin-embedded brain sections (black arrow, Fig. 5E). The subcellular distribution of SIRT1, p-JNK, and caspase-3 were studied using immunohistochemistry in post-mortem human brain tissues. The SIRT1 immunoreactivity was increased in CI brain, with immunoreactivity being detected from both nucleus and cytoplasm. Moreover, we observed an increase in p-JNK and caspase-3 immunoreactivity in stroke brain. Caspase-3 immunoreactivity was limited to the cytoplasm in control tissue, whereas, in stroke brain tissues, caspase-3 immunoreactivity was also observed in the nucleus (Fig. 5F–H). Further, the neurodegeneration in stroke brain was evidenced by immunofluorescence, wherein an increased number of Fluoro-Jade (FJ)-C positive cells within stroke brain was observed compared with the controls (Fig. 5I).

### SIRT1 activation with resveratrol modulates JNK/ERK/MAPK pathway following MCAO in aged rats

**Figure 6:**
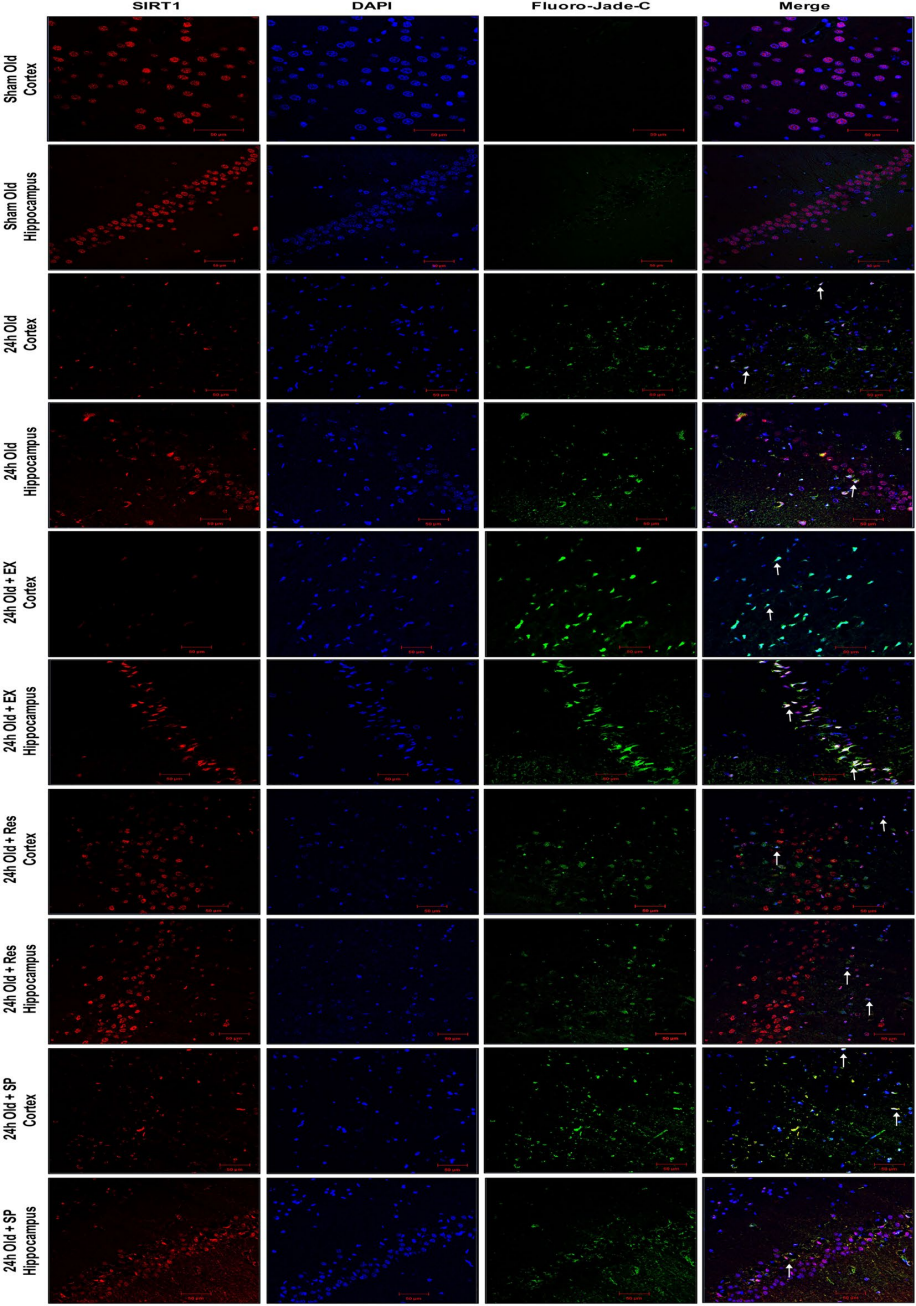
Immunofluorescence labeling of SIRT1 in brains of the aged experimental rat. Triple immunofluorescence staining is used to probe for SIRT1 (Red), DAPI (Blue), and Fluoro Jade-C (Green) to assess neurodegeneration in cortex and hippocampus neurons for control as well as brains from 24 h after MCAO, MCAO + EX-527, MCAO + resveratrol, and MCAO + SP600125 treatment. White arrows indicate co-localization of FJ-C with SIRT1 and DAPI (n = 4; Scale Bar—50 μm).

To understand the interaction of SIRT1 with JNK/ERK/MAPK pathway during neuroprotection, resveratrol treatment was given for activation of SIRT1 in MCAO rats. Our results demonstrated that activation of SIRT1 increased phosphorylation of MAPKs, including JNK, MEK, and ERK (p-MEK–p < 0.001, SIRT1-p < 0.004; Fig. 1G–J). The amount of infarct was measured using TTC staining, and reduction in infarct was not significant enough in resveratrol-treated ischemic aged rats compared with the MCAO group (Fig. 2D). Furthermore, increased immunoreactivity was seen against SIRT1 and p-JNK after resveratrol treatment compared to the MCAO group (Fig. 3A,B). Caspase-3 immunoreactivity was also assessed to confirm if the neuroprotective effect of resveratrol in aged experimental rats is associated with the downregulation of apoptosis. No significant decrease in caspase-3 immunoreactivity was observed (Fig. 3C). In this connection, there was no significant decrease in the number of FJ-C positive cells in the resveratrol-treated group compared with the MCAO group (Fig. 6).

**Figure 7:**
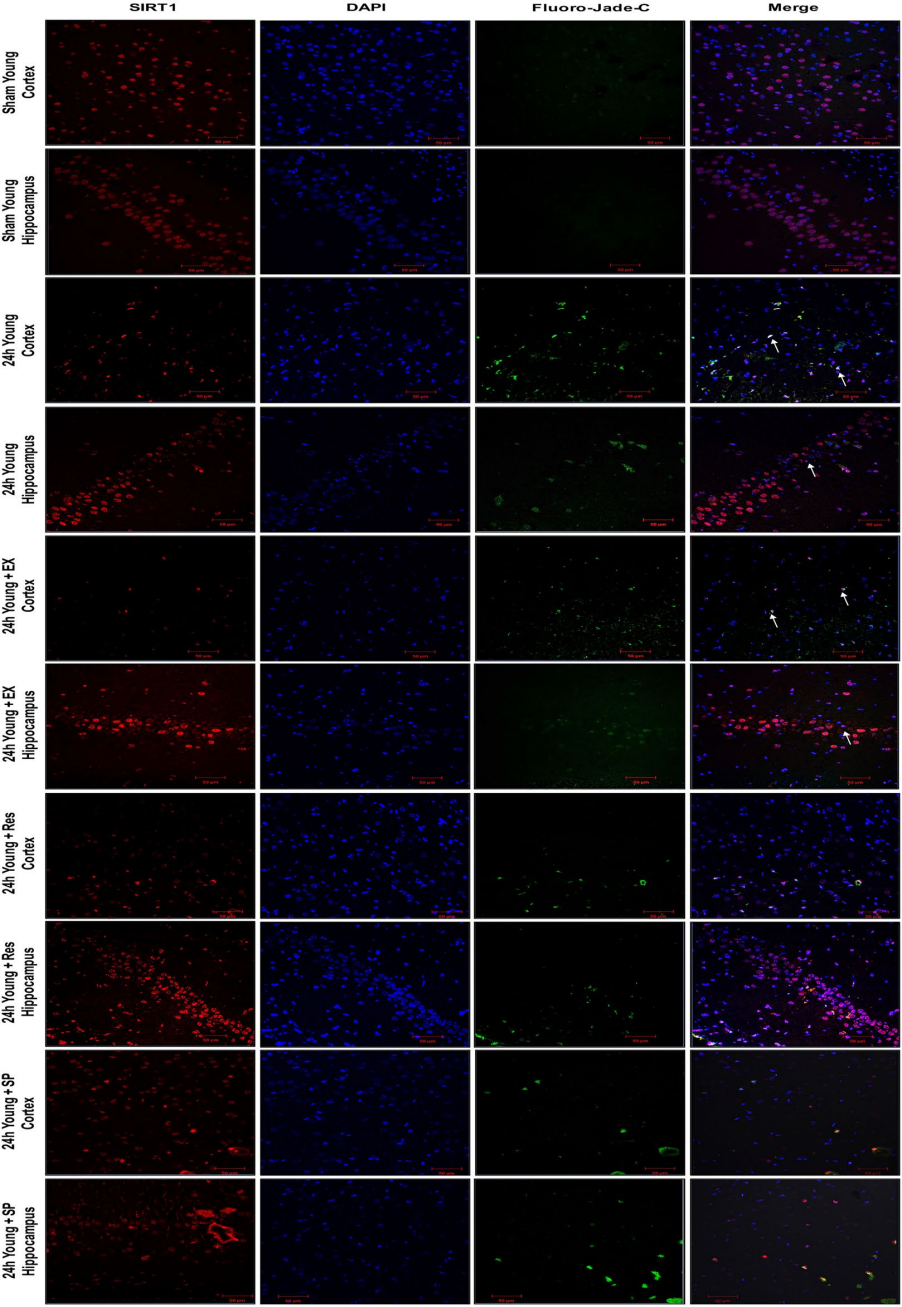
Immunofluorescence labeling of SIRT1 in brains of the young experimental rat. Triple immunofluorescence staining is used to probe for SIRT1 (Red), DAPI (Blue), and Fluoro Jade-C (Green) to assess neurodegeneration in cortex and hippocampus neurons for control as well as brains from 24 h after MCAO, MCAO + EX-527, MCAO + resveratrol, and MCAO + SP600125 treatment. White arrows indicate co-localization of FJ-C with SIRT1 and DAPI (n = 4; Scale Bar—50 μm).

Contrastingly, resveratrol conferred neuroprotection in young ischemic rats by up-regulation of SIRT1, and reciprocal down-regulation of JNK/ERK/MAPK (p-JNK–p < 0.001; p-ERK–p < 0.001; Fig. 1G–J), decreased neurodegeneration and caspase-3-mediated apoptosis (Figs. 4C, 7).

### SIRT1 inhibition with EX-527 decreases JNK/ERK/MAPK phosphorylation following MCAO in aged rats

The relation between SIRT1 and JNK/ERK/MAPK was further explored by inhibition of SIRT1 with EX-527. The pharmacological inhibition of SIRT1 significantly downregulated the phosphorylation of JNK/ERK/MAPK in EX-527-treated MCAO rats compared to the control (SIRT1–p < 0.001; p-JNK–p < 0.001; p-ERK–p < 0.001; p-MEK–p < 0.001, Fig. 1M–P). The immunoreactivity of SIRT1 was decreased following EX-527 treatment compared to the MCAO group (Fig. 3A). More importantly, the immunoreactivity of p-JNK decreased on SIRT1 inhibition, and neuronal injury was confirmed by increased caspase-3 immunoreactivity and increased FJ-C positive cells (Figs. 3B,C, 6). Thus, the downregulation of JNK/ERK/MAPK by SIRT1 inhibition proved that SIRT1 conferred neuroprotection by associating with JNK/ERK/MAPK in the aged ischemic rats.

On the contrary, SIRT1 inhibition with EX-527 in the brains of young ischemic rats significantly upregulated the phosphorylation of stress-responsive JNK/ERK/MAPK (SIRT1–p < 0.001; p-JNK–p < 0.001; p-ERK–p < 0.001; p-MEK–p < 0.001, Fig. 1 M–P), increased pro-apoptotic caspase-3, and augmented neuronal cell loss (Figs. 4C, 7).

### JNK inhibition with SP600125 aggravates neuronal damage following MCAO in aged rats

The synergistic relation between SIRT1 and JNK/ERK/MAPK and their neuroprotective nature in aged brains following CI was confirmed with the inhibition of JNK activity with SP600125 in aged MCAO rats. A significant decrease in MEK/ERK phosphorylation was observed compared to control (p-JNK–p < 0.001; p-MEK–p < 0.001; p-ERK–p < 0.002, Fig. 1T–V). The examination of the expression levels and subcellular localization of SIRT1 showed no substantial change in SIRT1 expression on SP600125 treatment compared with the brain tissues of stroke rats (Figs. 1S, 3A). The immunoreactivity of p-JNK was decreased in the presence of SP600125 compared with MCAO group (Fig. 3B). Importantly, caspase-3 immunoreactivity from the neuronal nuclei and the number of FJ-C positive cells were significantly increased on JNK inhibition in MCAO rats compared with control (Figs. 3C, 6). These results suggest that MAPK-associated neuroprotection in aged rats is mediated by SIRT1.

In contrast, inhibition of JNK activity in young MCAO rats during ischemia significantly decreased pro-apoptotic caspase-3 and neurodegeneration (Figs. 4C, 7).

### SIRT1 modulates IRS-1/AKT pro-survival pathway following MCAO in aged rats

The role of SIRT1/JNK axis on the phosphorylation status of IRS-1/AKT signaling was experimentally demonstrated. An increase in IRS-1 phosphorylation at Ser-307 and a decrease in AKT phosphorylation was observed in the brains of both young and aged experimental rats (p-IRS-1–p < 0.001; p-AKT–p < 0.001, Fig. 1E,F). More importantly, IRS-1 phosphorylation at Ser-307 was increased in aged rats following MCAO, despite a significant decrease in JNK phosphorylation. JNK inhibition decreased AKT phosphorylation but failed to affect the phosphorylation status of IRS-1 at Ser-307, which was contrary to the findings for young rats (p-AKT–p < 0.001; p-IRS-1–p < 0.001, Fig. 1W,X).

Inhibition of SIRT1 with EX-527 increased IRS-1 phosphorylation at Ser-307 and decreased AKT phosphorylation. Further, resveratrol treatment abolished the effect of EX-527 in both groups (p-AKT–p < 0.03; p-IRS-1–p < 0.001, Fig. 1K,L), (p-IRS-1–p < 0.001, Fig. 1Q,R). These results demonstrated that activation of the AKT pro-survival pathway is dependent on SIRT1.

## Discussion

SIRT1 is a mediator of caloric restriction-induced longevity and known for protection against aging^23^. SIRT1 has been implicated in neuronal development, learning, and memory^5,18^. Emerging evidence supports that genetic and pharmacological manipulation of SIRT1 modulates stroke outcome. Reduced neuronal damage is incurred by SIRT1-transgenic mice owing to SIRT1 overexpression^24^. Another line of evidence indicates that activation/inhibition of SIRT1 with pharmacological agents modulates CI outcome^25^. A similar effect was observed for ischemic preconditioning and resveratrol preconditioning. In both paradigms, SIRT1 activation protects from ischemic neuronal injury^26,27^. Recent findings in the human population reported that serum levels of SIRT1 are significantly higher in patients with acute IS ^28^. In relation with this, our findings are consistent with those of previous reports. We observed decreased SIRT1 expression in the brains of experimental rats following CI and increased SIRT1 expression in patients with stroke.

However, recent data show that under specific settings, SIRT1 inhibition, rather than its activation, is neuroprotective^22,29,30^. The contradictory results indicate that subcellular localization of SIRT1 may play a role in the regulation of cell death. SIRT1 is highly expressed in the brain and spinal cord during embryogenesis. Also, it is express in the adult brain occurring at high levels in cortex, hippocampus, cerebellum, and hypothalamus and low levels in white matter^31^. SIRT1, a histone deacetylase, is customarily localized in the nucleus^32^. However, studies have confirmed that it is also localized in the cytoplasm^22,33^. Cytosol-localized SIRT1 may function as pro-apoptotic factor^34^, whereas nuclear-localized SIRT1 could possibly act as anti-apoptotic factor^35,36^. Our study results showed that SIRT1 is highly expressed in the cortex and hippocampus. In control brains, SIRT1 immunoreactivity was predominantly from the nucleus. Still, in the infarcted brain, immunoreactivity was also observed in the cytoplasm, which may provide a possibility for the effect of SIRT1 on cytosolic MAPKs activation following CI.

Caspase-3 is believed to be one of the vital effector caspases involved in the apoptotic cell death in hypoperfused peri-infarct area in experimental models and human stroke brains^37,38^. One of the survival mechanisms thought to be regulated by SIRT1 following CI is inhibition of caspase-3 dependent apoptosis^39^. In the present study, results showed that SIRT1 inhibition with EX-527 increased caspase-3, suggesting that SIRT1 activity is necessary for neuronal survival following CI. Our results have consolidated the idea that inhibition of SIRT1 with EX-527 significantly increased neuronal apoptosis^39^.

However, studies proclaim that administration of resveratrol is neuroprotective following CI^40^. Resveratrol treatment enhances neuronal survival and reduces caspase-3 mediated apoptosis via SIRT1 activation in subarachnoid hemorrhage experimental rat model^41^. Further, resveratrol promotes adult neurogenesis in the subventricular zone and hippocampus via the upregulation of SIRT1 expression^42^ and resveratrol pretreatment protects CA1 hippocampal neurons in SIRT1-dependent manner^27^. In our study, resveratrol treatment failed to show a significant enhancement in protecting the aged brain from caspase-3 mediated neuronal apoptosis. The diminished response to acute resveratrol treatment in aged rats is attributed to age-induced increase in apoptosis^43^ and the properties of resveratrol per se. Acute resveratrol treatment following CI modulates multiple cell-signaling pathways that might have a detrimental effect on cell survival^44,45^. Resveratrol adversely affects hippocampal neurogenesis and cognitive function via AMPK activation and inhibition of cAMP response element-binding protein (CREB), brain-derived neurotrophic factor (BDNF) signaling^46^. More importantly, resveratrol is proven to increase oxidative stress in the brain of the adult mouse by binding to mitochondrial complex-I^47^.

Increasing evidence suggests that CI triggers various stress-responsive pathways that converge on MAP kinase family proteins. It is evident that MAPKs, including ERK/JNK/MAPK, are highly expressed in rat brain following CI. Pharmacological inhibition of JNK activity has been involved in postischemic neuroprotection in young experimental stroke models^48–50^. Although the neuronal loss in models of ischemia could be achieved by inhibition of MEK, the upstream activator of ERK. Recent studies demonstrated that ERK is an essential component in learning and memory formation^51,52^. However, the role of ERK in neuronal survival depends upon the intensity of the stress stimuli, cellular environment, and the duration of ERK activation^53^.

Recent studies demonstrated that under certain contexts, MAPK signaling cascade activation, rather than inhibition, is neuroprotective. SIRT1 upregulation protects against hepatic ischemia through overexpression of MAPKs and the protection conferred by SIRT1 is effectively reversed by the inhibition of MAPK family proteins^20^. The protective nature of JNK in cardiomyocytes and against ischemic preconditioning is well documented^19,54^. Recent studies in CI and traumatic brain injury (TBI) demonstrated that resveratrol-induced protective effects are mediated by SIRT1/MAPK signaling cascade^44,55^. Moreover, SIRT1 inhibition reduces ERK and protects neurons from oxidative stress^22^, suggesting the role of SIRT1 expression in MAPK signaling cascade activation in promoting neuronal survival.

In the present study, the phosphorylation of JNK/ERK/MAPK was decreased in aged experimental rats, and inhibiting JNK phosphorylation significantly increased the expression of caspase-3. Interestingly, JNK inhibition had no effect on SIRT1 expression. Our results are in disagreement with the findings of previous studies that JNK modifies the expression of SIRT1^56^. Further SIRT1 inhibition decreased JNK/ERK/MAPK phosphorylation. Nevertheless, our results in young experimental rats were congruent with previous reports. An inconsistency in SIRT1/MAPK signaling between young and aged experimental rats may depend on various factors involved in regulating SIRT1 functional activity, including intensity of the stress stimuli, context-dependent changes in molecular pathways^43,57^, availability of NAD^+^ pool^31^. Moreover, SIRT1 functional activity is reported to be decreased during aging. High levels of nicotinamide and lower levels of SIRT1 leads to further inhibition of SIRT1 functional activity in aged cells^58^. Accordingly, the activity of JNK/ERK/MAPK was upregulated in human patients. Together, these data support that SIRT1 synergistically interacts with MAPKs to regulate neuronal apoptosis.

IRS-1/AKT and SIRT1/JNK represent two parallel pathways and interaction between these two pathways could be either positive or negative. JNK phosphorylates IRS-1 at Ser-307, thereby negatively regulating AKT^59^. AKT activation by SIRT1 is necessary for its phosphorylation^18,60^. Activation of AKT promotes neuronal survival following CI^61^. Recent studies suggest the correlation between MAPKs and AKT activation in SIRT1-dependent manner in different settings^44,62^. Here, our study demonstrated that SIRT1 promotes AKT activation via MAPKs in experimental rats following CI and human patients.

In conclusion, our study indicates that SIRT1 modulates JNK/ERK/MAPK and AKT signaling cascade in experimental rats and human brains following CI. Inconsistency in the SIRT1/MAPK signaling cascade in experimental rat models and humans may be due to various pathways and mechanisms that are mediated by SIRT1 and MAPKs and the context-dependent changes in these pathways. However, further experimental studies are needed for a better understanding of SIRT1/MAPK/AKT signaling cascade and its modulation on stroke outcome, which indicates a profound application value.

## Materials and methods

### Transient middle cerebral artery occlusion (MCAO)

All the animal studies were carried out as per the requirement and guidelines of the Committee for the Purpose of Control and Supervision of Experiments on Animals (CPCSEA), Government of India and after obtaining permission of the Institutional Animal Ethics Committee (IAEC), University of Hyderabad (UH/IAEC/PPB/2018-I/27). All the experimental methods were carried out in compliance with the ARRIVE checklist^63^. Young (3–4 months) and aged (18–20 months) male Sprague–Dawley (SD) rats were procured from the National Centre for Laboratory Animal Sciences (NCLAS), India. Animals were housed under controlled environment with free access to water and food. Rats were randomly divided into five groups: (i) sham group (Control), (ii) MCAO group, (iii) MCAO + EX-527 group (intracerebroventricular (ICV) route; 30 μg/kg; before 30 min of MCAO)^39^, (iv) MCAO + resveratrol group (intraperitoneal (IP) route; 20 mg/kg; after 30 min of MCAO)^64^, (v) MCAO + SP600125 group (ICV route; 30 μg /kg; before 30 min of MCAO)^50^. All the drugs were purchased from Sigma (USA). All efforts were made to minimize the number of animals to be used.

Focal CI was produced by middle cerebral artery occlusion (MCAO), which was adopted as per the protocol of Longa et al^65^. Briefly, rats were anesthetized with ketamine (60 mg/Kg) and xylazine (10 mg/Kg) administration via the IP route. The middle neck incision was made to locate arteries mainly the left common carotid artery (CCA), internal carotid artery (ICA), and the external carotid artery (ECA). Microvascular clips were temporarily placed on CCA and ICA, while ECA was ligated distally. A 3–0 nylon monofilament was inserted from ECA by arteriotomy and gently advanced into ICA to block the origin of MCA. The occlusion of MCA origin was felt through the resistance created. For the sham-operated group, monofilament was inserted into CCA, but MCA was not occluded. After 60 min of occlusion, monofilament was removed gently to allow reperfusion, and the animals were allowed to recover.

### Neurological deficit evaluation

Neurological deficit was evaluated for infarct intensity using Bederson score^66^. Rats with no observed behavioral deficiency were scored as grade 0, rats which failed to extend forelimb contralateral to infarct were scored as grade 1, rats with decreased resistance to lateral push with infrequent circling toward ipsilateral side were scored as grade 2, rats with hemiparesis were scored as grade 3, and rats who died because of severe lesion were scored as grade 4. Animals with neurological scores below grade 3 were excluded from experimental groups.

### Infarct volume measurement

Brains were cut into six coronal slices with 2 mm thickness and stained with 2% 2,3,5- triphenyl tetrazolium chloride (TTC) (Sigma-Aldrich, US) at 37° C for 30 min, followed by 4% paraformaldehyde (PFA) fixation. Sections were photographed and analyzed with ImageJ software (NIH, US). Infarct areas from all segments were added to derive the total infarct, which was multiplied by the thickness of the brain section to determine total infarct volume. Correction for edema of infarct was performed as described previously^67^.

### Immunoblotting

Rats were decapitated with an overdose of pentobarbital at 24 h after reperfusion. Ipsilateral brains were excised and homogenized in RIPA buffer (150 mM NaCl, 2 mM ethylenediaminetetraacetic acid (EDTA), 50 mM Tris–HCl (pH-7.4), 10% glycerol, 1% NP40, and 0.5% sodium deoxycholate). Protein concentration in the supernatant was determined by the Bradford method following centrifuging tissue homogenates at 10,000 rpm for 20 min. An equal amount of total protein sample (75 μg) was probed by western blots. Total protein extract was resolved on 8% and 10% SDS-PAGE gels and transferred on to nitrocellulose membrane. The membranes were incubated with 5% nonfat dry milk (HIMEDIA, India) in tris-buffered saline containing Tween-20 (TBS-T) for 90 min at room temperature (RT). The membranes were incubated overnight at 4 °C with primary antibody (1:1000 dilution) against SIRT1, phospho and total-JNK, phospho and total-ERK, phospho and total-MEK, phospho and total-AKT, beta-tubulin (Cell Signaling Technology, USA), and phospho-IRS-1 (ser307) (Merck, USA). Membranes were washed with TBS-T and incubated with HRP labeled secondary antibody (CST, USA) specific for mouse and rabbit for 90 min at 37 °C (1:3000 dilution). The chemiluminescence was captured using photographic film and ECL detection system (Bio-Rad, USA).

### Histology and immunohistochemistry

Brains tissues at 24 h after reperfusion were fixed by trans cardiac perfusion with phosphate-buffered saline (PBS) and 4% paraformaldehyde (PFA). Brains tissues embedded in paraffin were sectioned into slices having 5–10 μm thickness using the microtome (Leica, Germany). Brain sections were stained with hematoxylin and eosin (H&E) and then immunostained using an immunohistochemistry detection kit (Bio SB, USA) against SIRT1, p-JNK, and caspase-3. Sections were dewaxed in three changes of xylene each for 5 min, then hydrated in a decreasing gradient of alcohol for 5 min each. Endogenous peroxidase was inhibited by quenching sections with 3% hydrogen peroxide in methanol, followed by heat-induced antigen retrieval in 10 mM citrate buffer with 0.05% tween-20 (pH-6.0). After cooling, sections were covered with 0.25% BSA for 30 min to minimize nonspecific binding. Sections were treated with primary antibody (1:100 dilution) in blocking solution for 2 h 30 min at RT, followed by HRP-labelled secondary antibody for 45 min at RT (provided with the kit). Staining was visualized by covering the sections in buffer containing 3′-3′ diaminobenzidine (DAB) (supplied in kit) for 5 min, counterstained by hematoxylin counterstaining. After dehydration, sections were mounted and images were captured under the light microscope (Olympus, Japan) with 400 X magnification.

### Immunofluorescence

Triple immunofluorescence labeling was performed to probe for SIRT1, DAPI (CST, US), and Fluoro Jade-C (FJ-C), (Millipore, US), which stains degenerating neurons. Sections from both experimental rats and humans were deparaffinized and rehydrated through 100–70% graded ethanol in distilled water. Sections were covered in antigen retrieval solution (citrate buffer) and boiled three times, each for 5 min with 1 min interval in a microwave. Sections were blocked by 5% goat serum for 60 min at 37 °C and incubated in anti Sirt1primary antibody for overnight at 4 °C. After being washed with PBS, sections were incubated with Alexa Fluor-555 conjugated goat anti-mouse IgG (CST, US) for 60 min at room temperature. After PBS washing, sections were covered with 0.06% KMnO_4_ for 10 min, followed by Fluoro Jade-C incubation for 30 min at 37 °C. Slides were mounted with antifade DAPI, and images were captured under the laser scanning confocal microscope (Carl-Zeiss, Germany).

### Post-mortem human brain tissue

Autopsied ischemic human brain tissues (n = 10) were obtained from “Human Brain Bank, Department of Neuropathology, National Institute of Mental Health & Neurosciences (NIMHANS); Bangalore, India.” Informed consent to commence autopsy was obtained from family members of the patient. All the experimental protocols were approved by the Institutional Ethical Committee (IEC), University of Hyderabad, India-(UH/IEC/2019/92). All methods were performed in accordance with relevant guidelines and regulations. Healthy brain tissue without any neurological abnormality was used as control (n = 3). Patient details including age, sex, and region of infarct are given in Table 1. Brain tissue was homogenized in RIPA buffer using the same method stated in the immunoblotting section. Total protein extract was separated on 10% SDS-PAGE gels and antibody used against SIRT1, phospho and total JNK, phospho and total ERK, phospho and total AKT, and beta-actin. Chemiluminescence was visualized using the ECL detection system (Bio-Rad, USA). Brain tissues were embedded in paraffin following formalin fixation. Formalin-fixed brains were sectioned into 5–10 μm-thick slices and stained with H&E to assess morphological changes. Immunohistochemistry was performed against SIRT1, p-JNK, and caspase-3 using the method as mentioned in the immunohistochemistry section.

**Table 1:** Summary of the patient’s information.

Case	Age/sex	PMI	Anatomical area
1	42/M	2 h 30 min	Orbito frontal cortex (Normal)
2	44/M	19 h 15 min	Insular (Normal)
3	35/M	12 h	Left orbito frontal (Normal)
4	35/M	12 h	Right orbito frontal infarct
5	43/M	4 h	Right MCA territory infarct
6	65/F	12 h 30 min	Necrotic tissue right infarct
7	23/F	16 h 40 min	Hemorrhagic infarct
8	35/F	24 h	Right parieto-occipital infarct
9	19/F	NA	Left frontal infarct
10	25/F	5 h	Left temporal infarct
11	25/F	NA	Right temporal infarct
12	38/F	15 h 15 min	Right temporal infarct
13	22/F	NA	Right fronto parietal infarct

*MCA* middle cerebral artery, *M* male, *F* female, *PMI* post-mortem interval.

### Statistical analysis

All the experimental rat data expressed as the mean ± SD and analyzed for statistical significance using one-way ANOVA with Tukey’s multiple comparisons test, and human data were subjected to paired t-test. Graphs were plotted with GraphPad Prism 7.0 software (GraphPad Inc. USA). Bars represent variation in the experimental samples. Significance was shown as asterisks: * indicates p < 0.05, while p > 0.05 indicates no significance (ns).

### Ethical approval & consent to participate

All human brain tissue samples were obtained from Brain Bank—NIMHANS with appropriate ethical approval from Institutional Ethics Committee (IEC)—University of Hyderabad. UH/IEC/2019/92. All methods were performed in accordance with relevant guidelines and regulations. All the animal studies were carried out as per the requirement and guidelines of the Committee for the Purpose of Control and Supervision of Experiments on Animals (CPCSEA), Government of India and after obtaining permission of the Institutional Animal Ethics Committee (IAEC), University of Hyderabad (UH/IAEC/PPB/2018-I/27). All the experimental methods were carried out in compliance with the ARRIVE checklist.

## Supplementary information

Supplementary figures.

## Data Availability

All the data generated or analysed during this study are included in this article along with its supplementary information.
