# Supplementary material for: Differential role of SIRT1/MAPK pathway during cerebral ischemia in rats and humans
Source: Sci Rep. 2021 Mar 18;11:6339. doi: 10.1038/s41598-021-85577-9 (PMC7973546; doi:10.1038/s41598-021-85577-9)

Differential role of SIRT1/MAPK pathway during cerebral ischemia in rats and humans

Sireesh Kumar Teertam[1] (Ph.D.), and Phanithi Prakash Babu (Ph.D.) *

Department of Biotechnology and Bioinformatics, School of Life Sciences, University of Hyderabad, Hyderabad (T.S), India.

*Corresponding author: Phanithi Prakash Babu. Department of Biotechnology & Bioinformatics, School of Life Sciences, University of Hyderabad, Prof. C. R. Rao Road, Gachibowli, Hyderabad -500 046 (T.S), India:

E-mail & phone: prakash@uohyd.ac.in; +91-40-23134584.

Figure-1 Supplementary: **24 h MCAO**

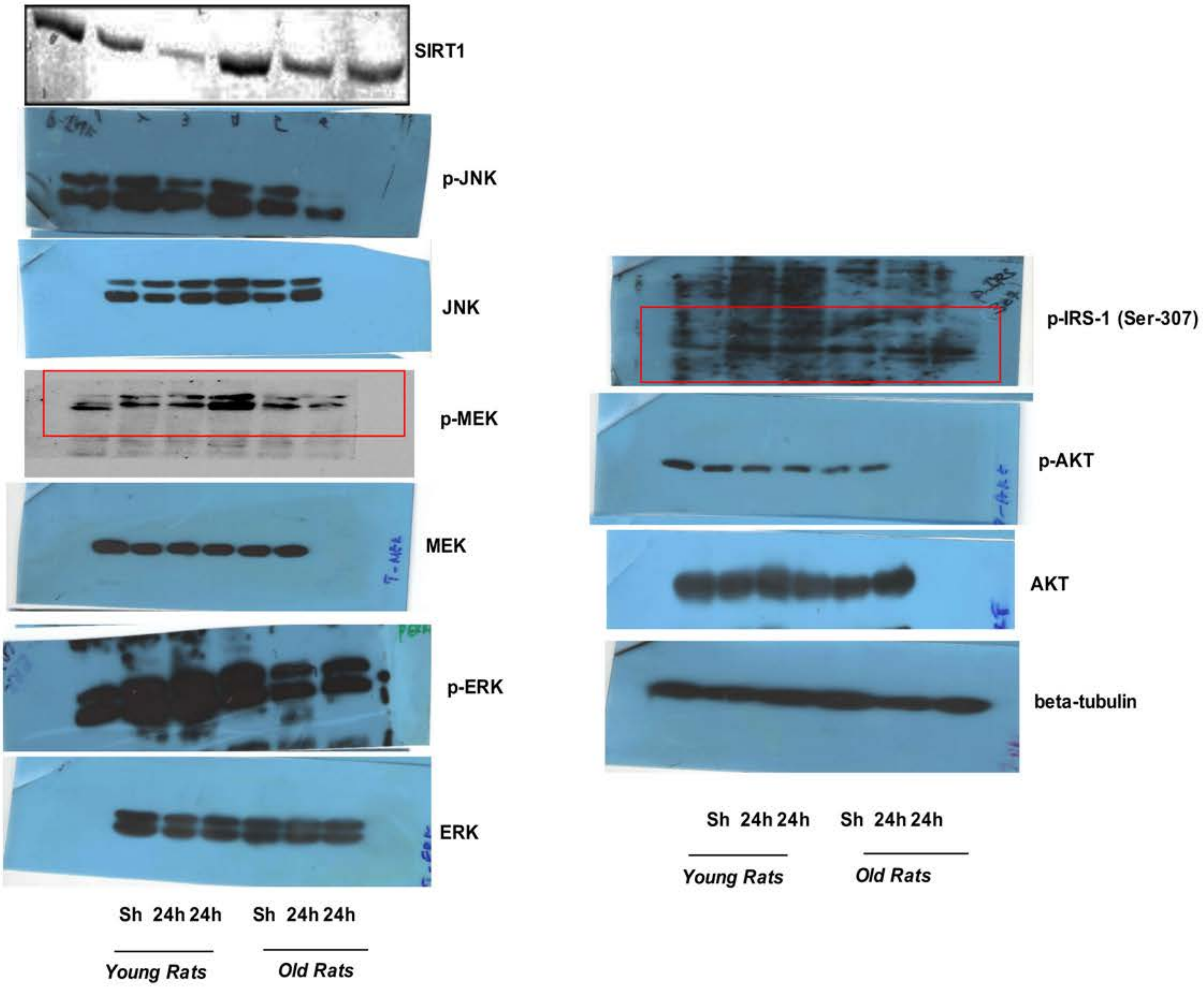

Figure-1 Supplementary: **24 h MCAO + Resveratrol**

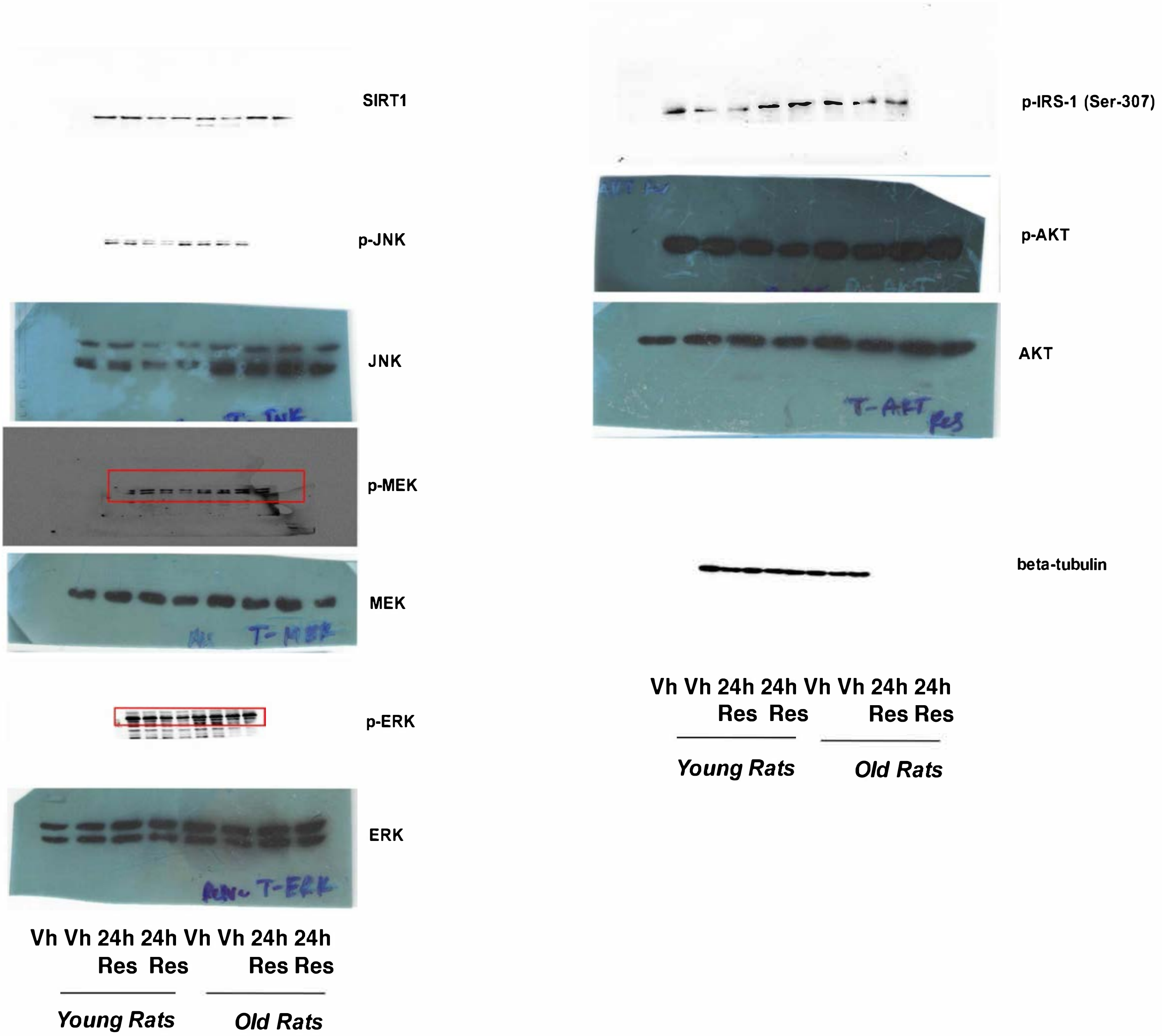

Figure-1 Supplementary:24 **h MCAO + EX-527**

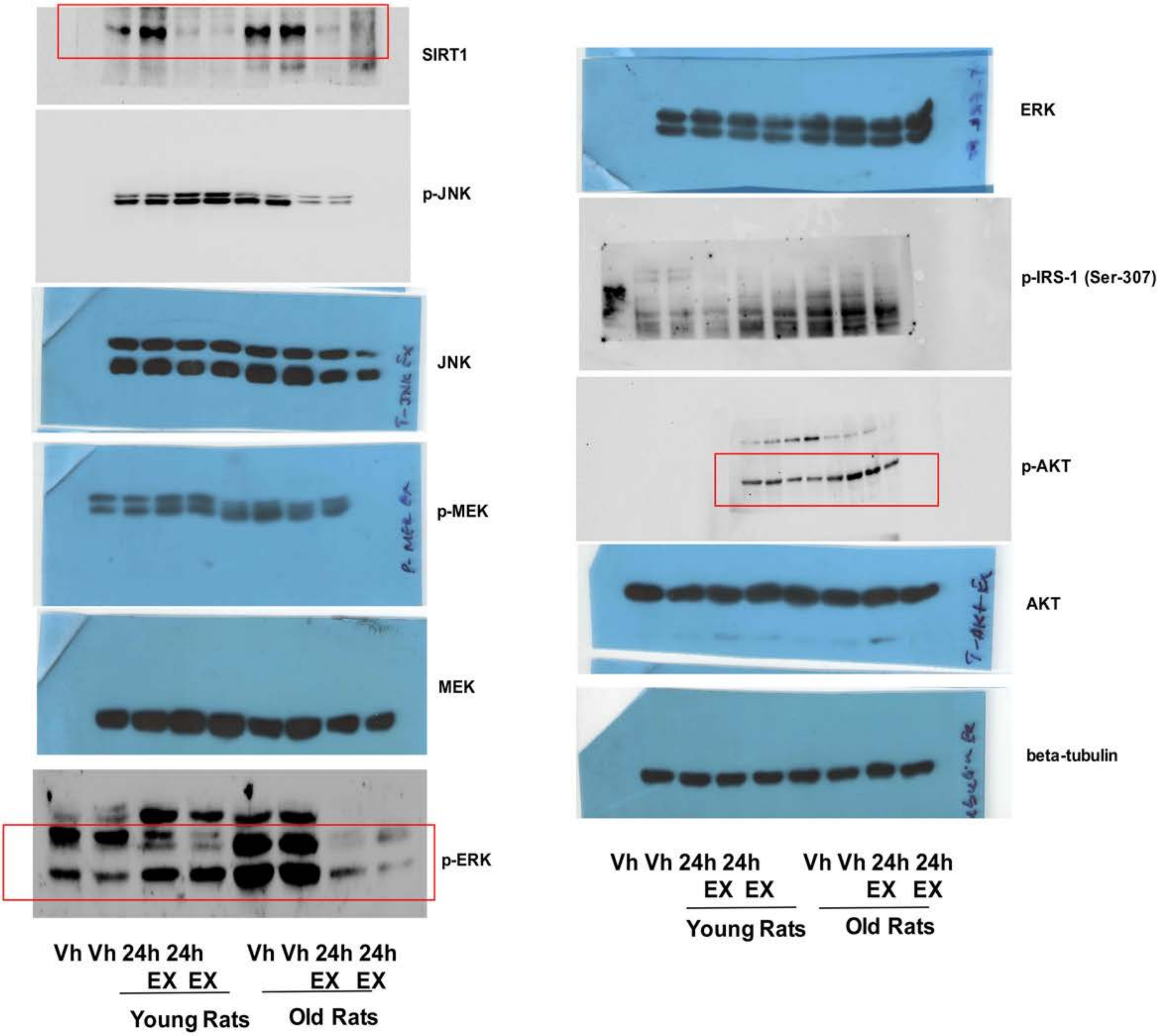

Figure-1 Supplementary: **24 h MCAO + SP600125**

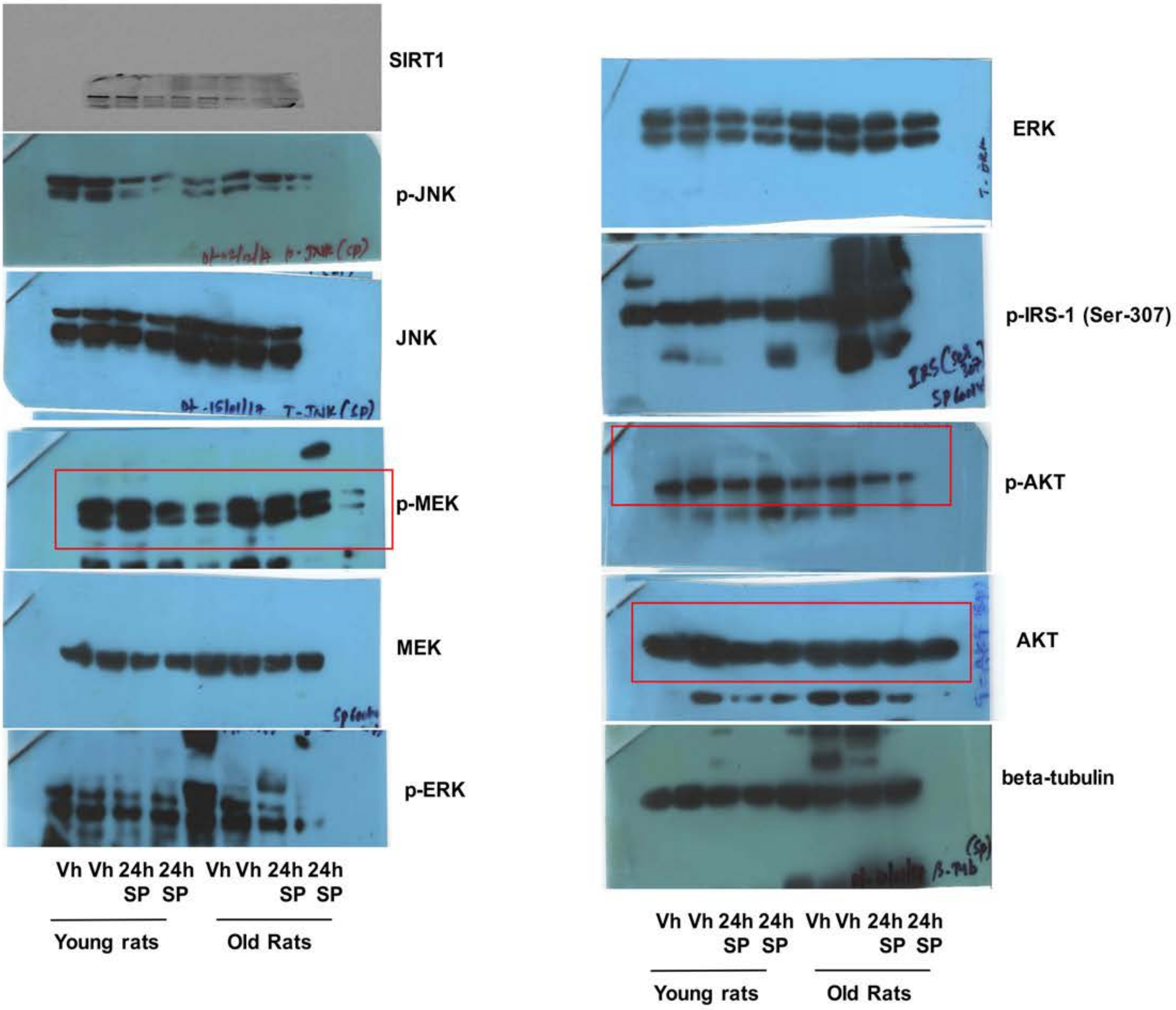

Figure-2 Supplementary: Hematoxylin & Eosin staining

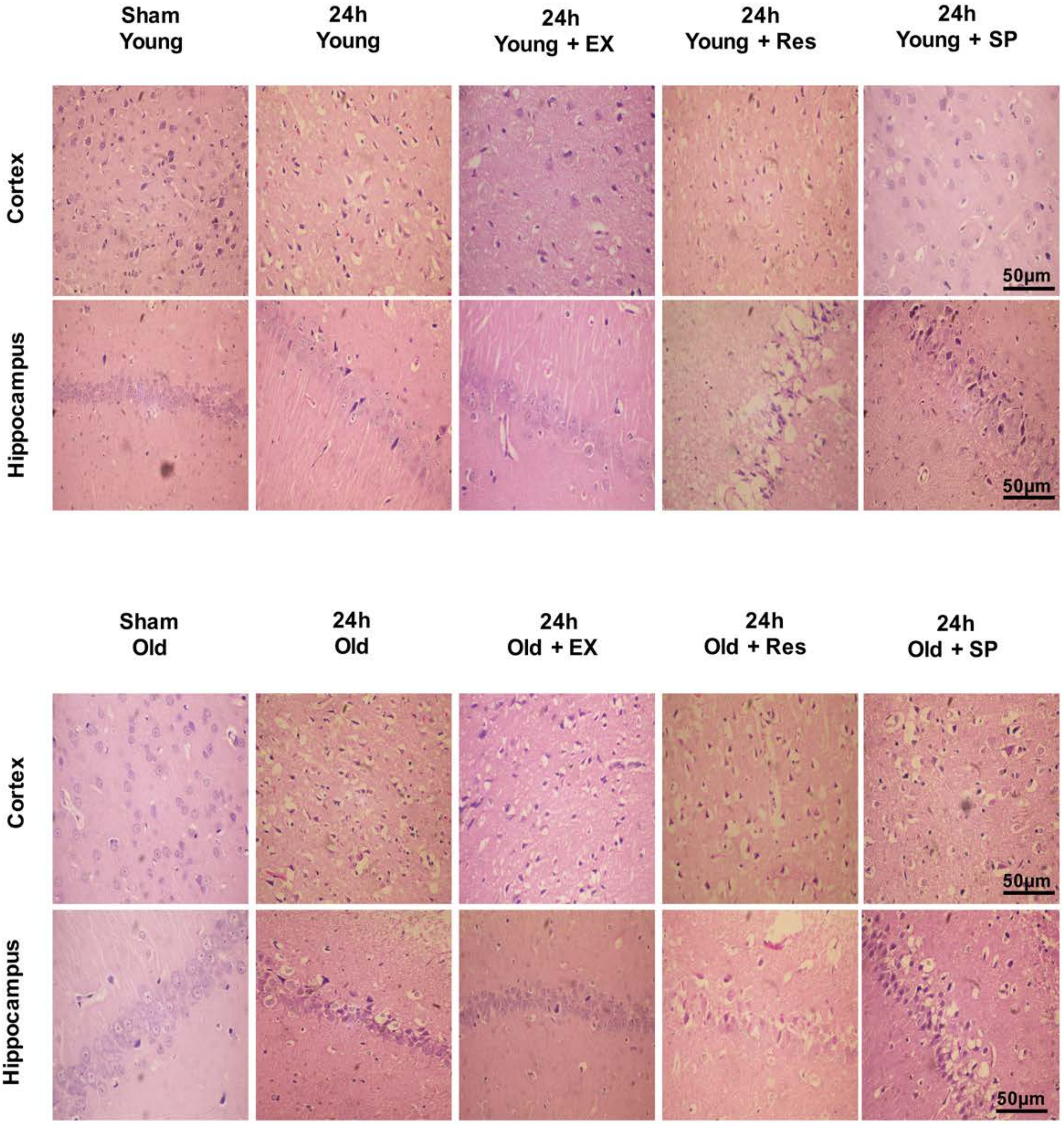

Figure-2 Supplementary: TTC staining

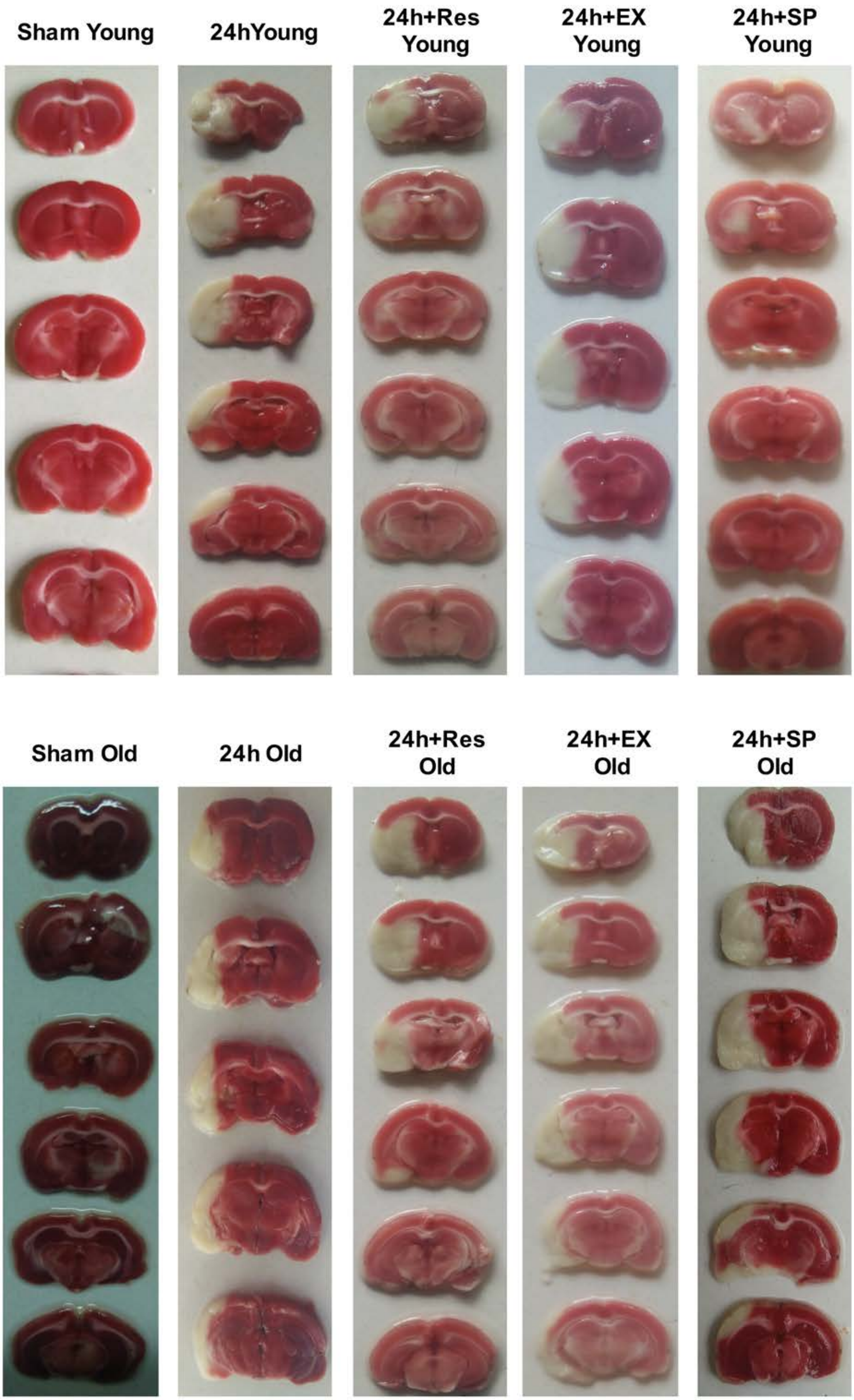

IHC in Aged Rats Supplementary

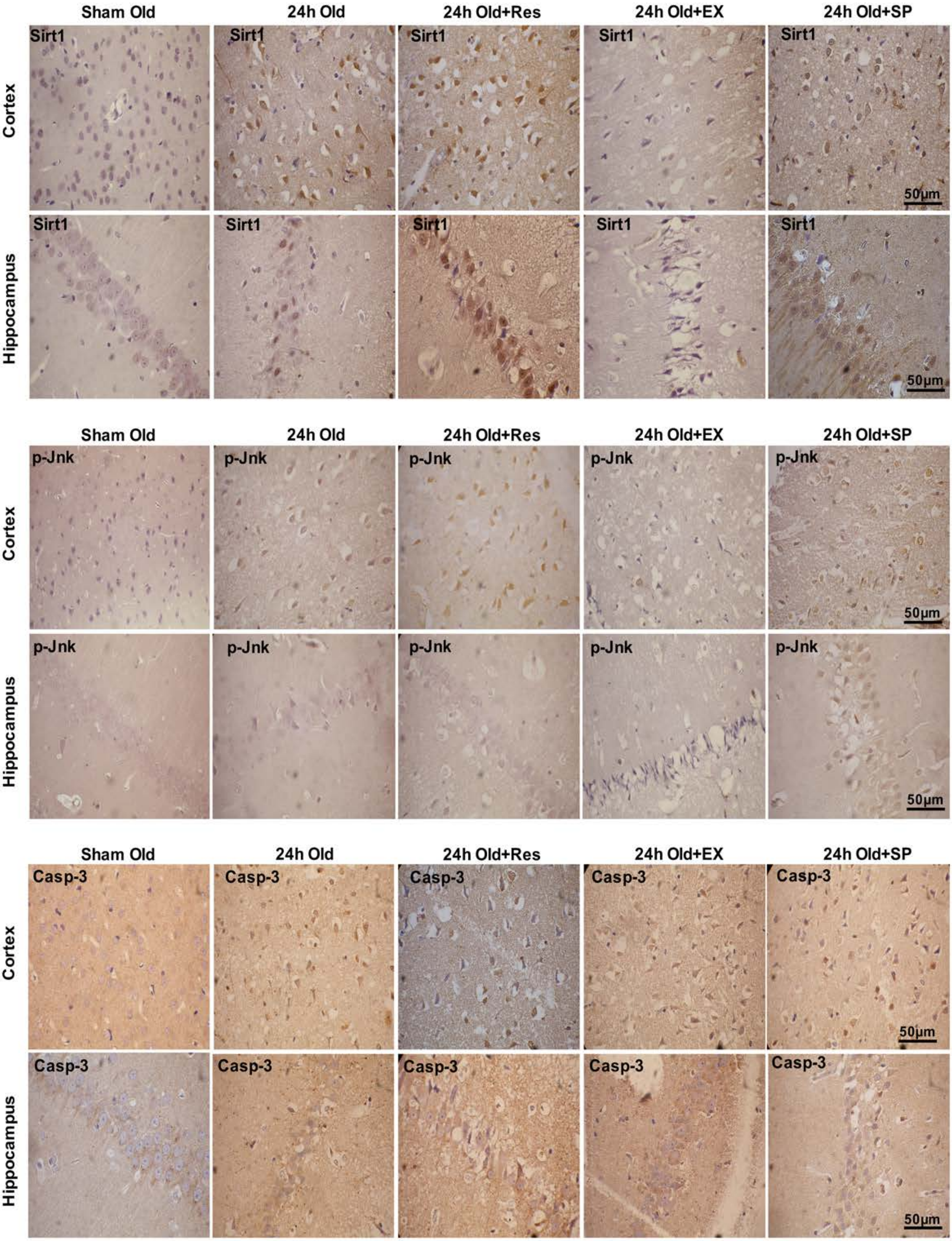

Figure-3 supplementary data: Immunohistochemistry data quantification for SIRT1, p-JNK, and caspase-3 in aged experimental rats.

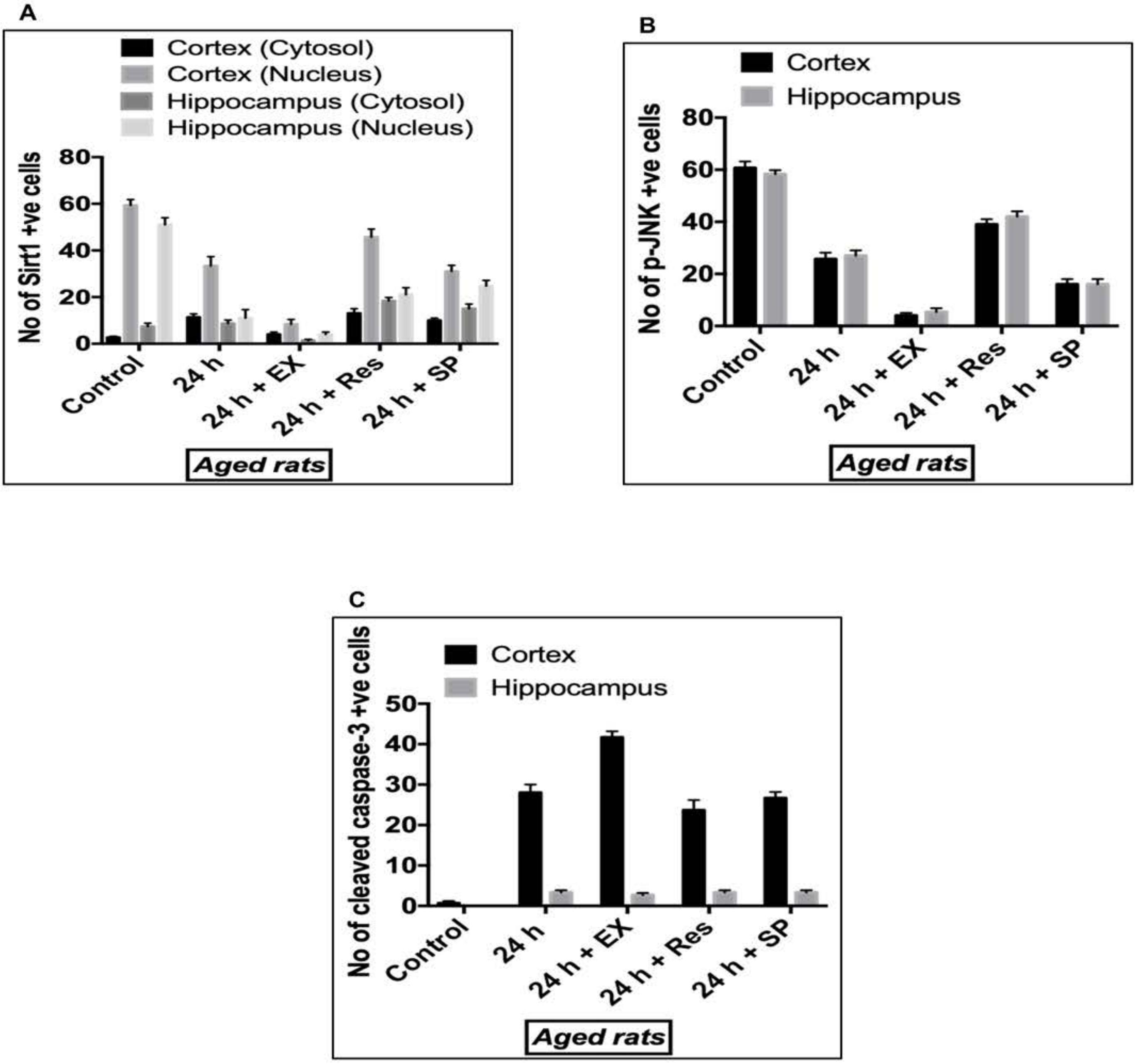

IHC in Young Rats Supplementary

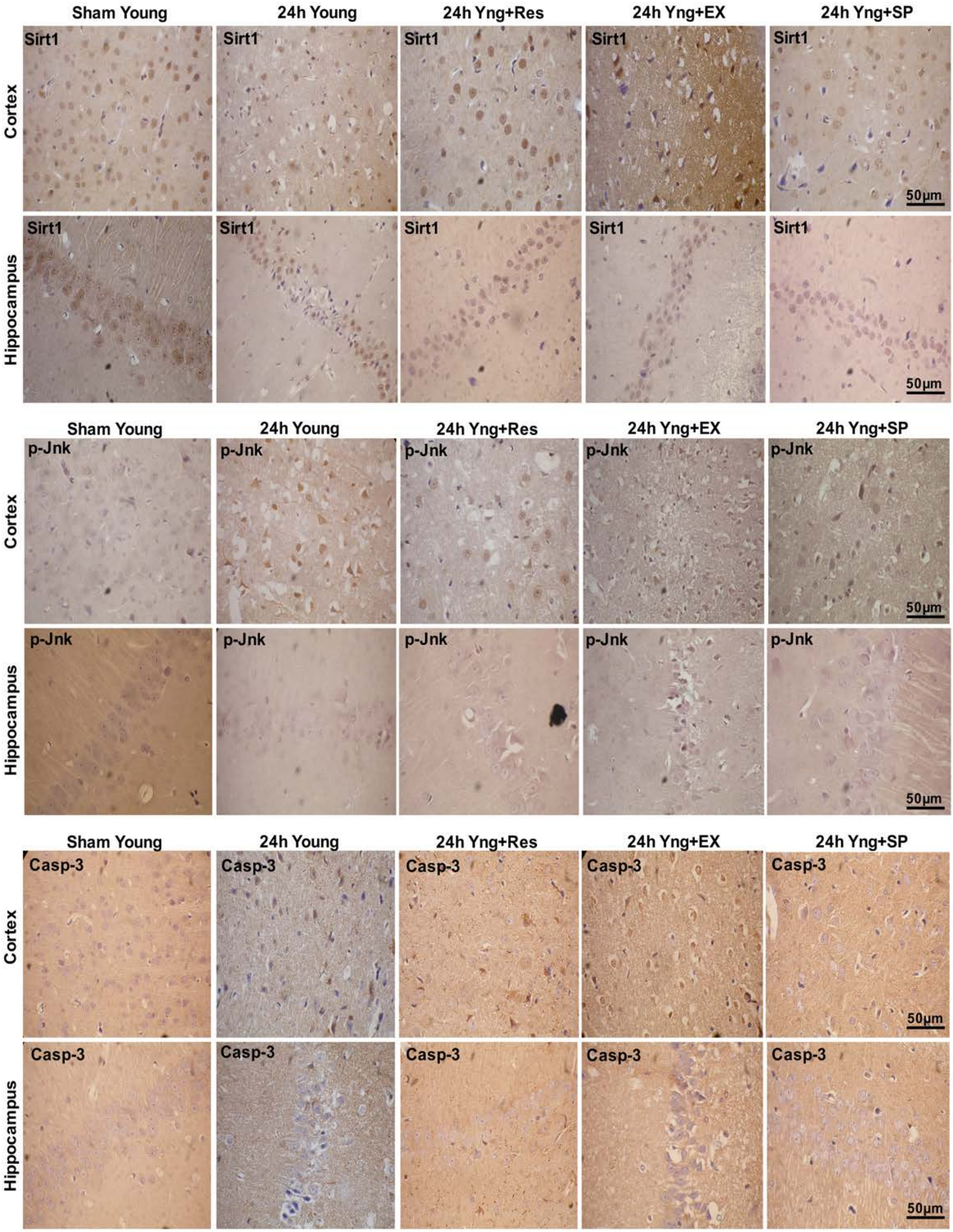

Figure-4 supplementary data: Immunohistochemistry data quantification for SIRT1, p-JNK, and caspase-3 in young experimental rats.

A

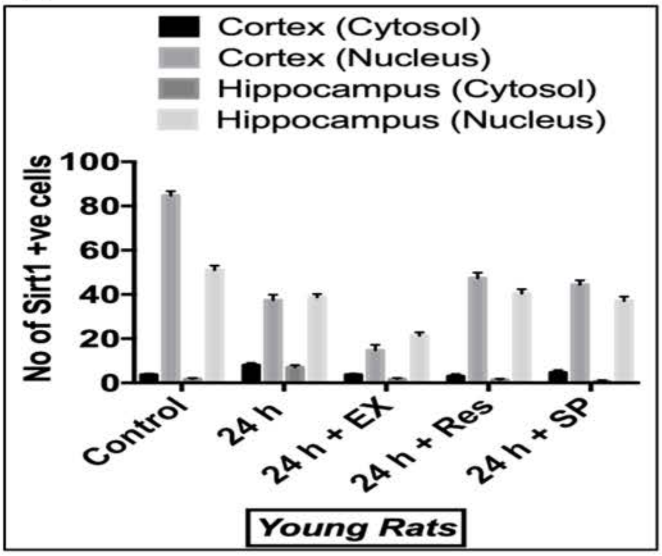

B

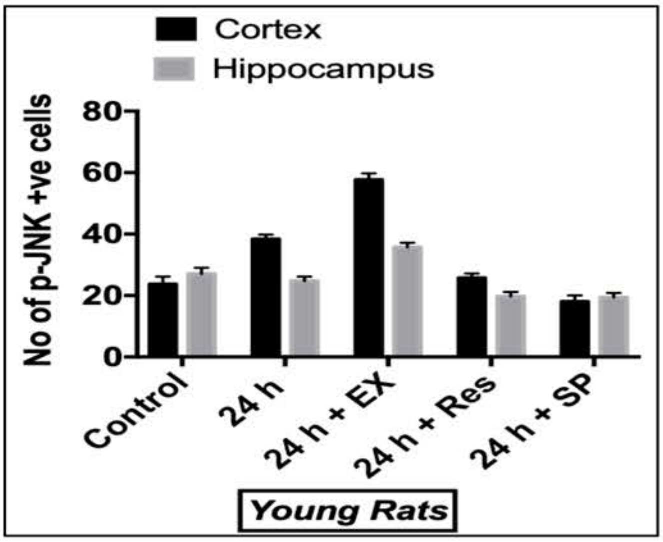

C

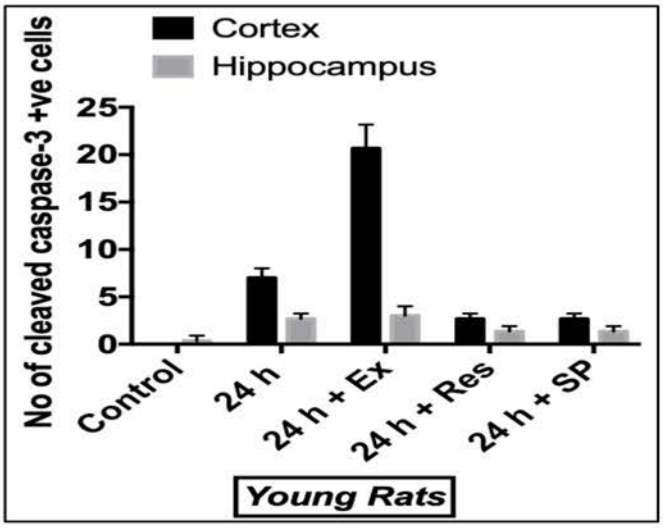

Human Supplementary

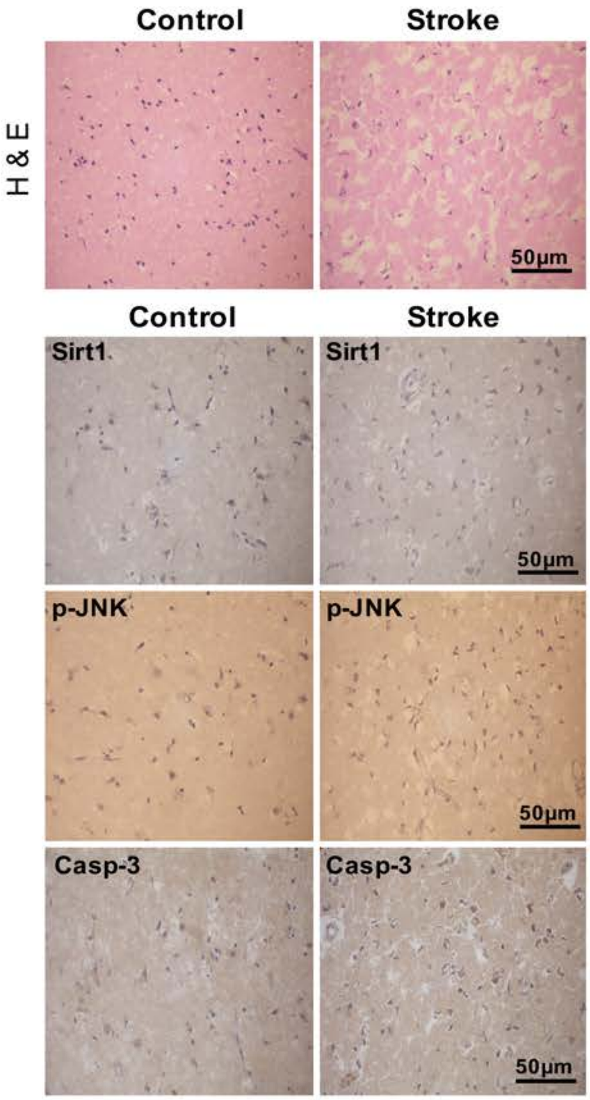

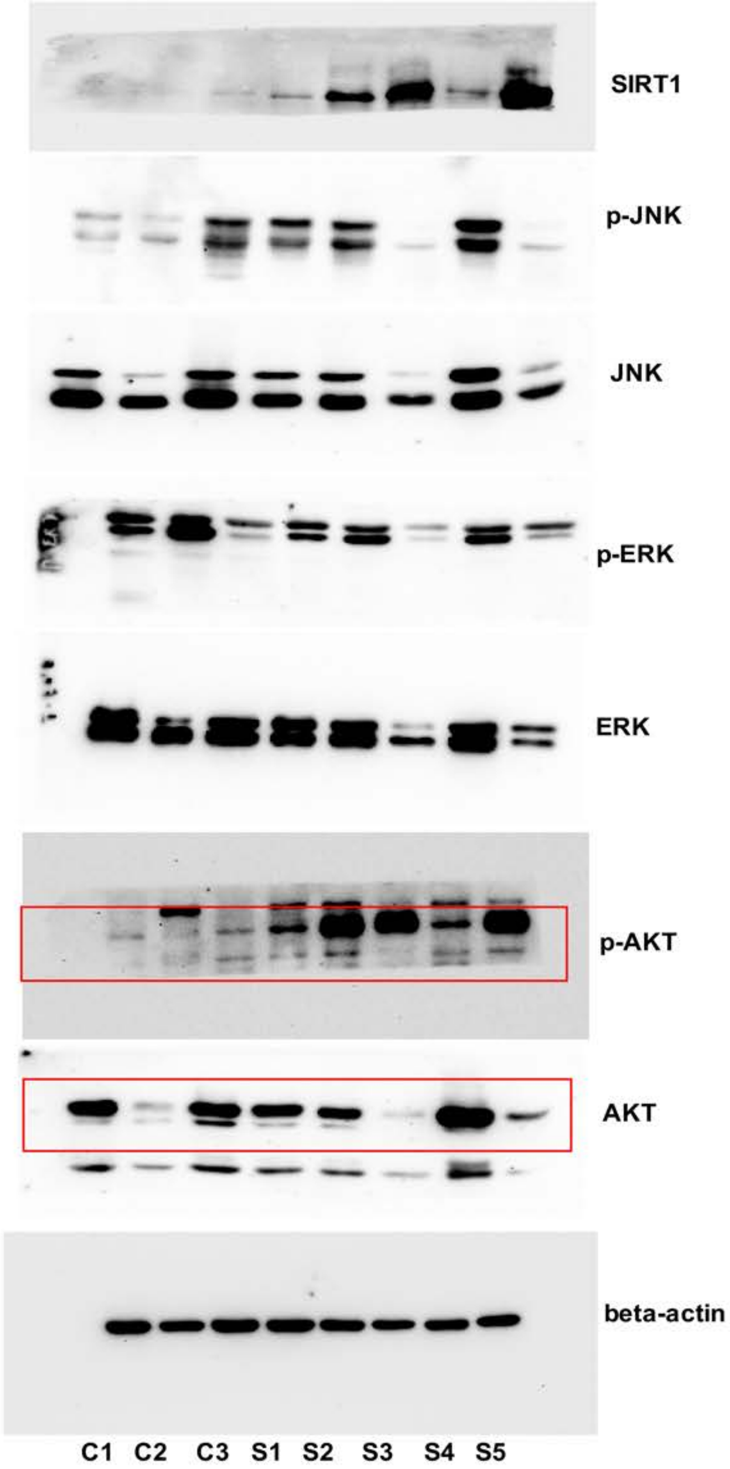

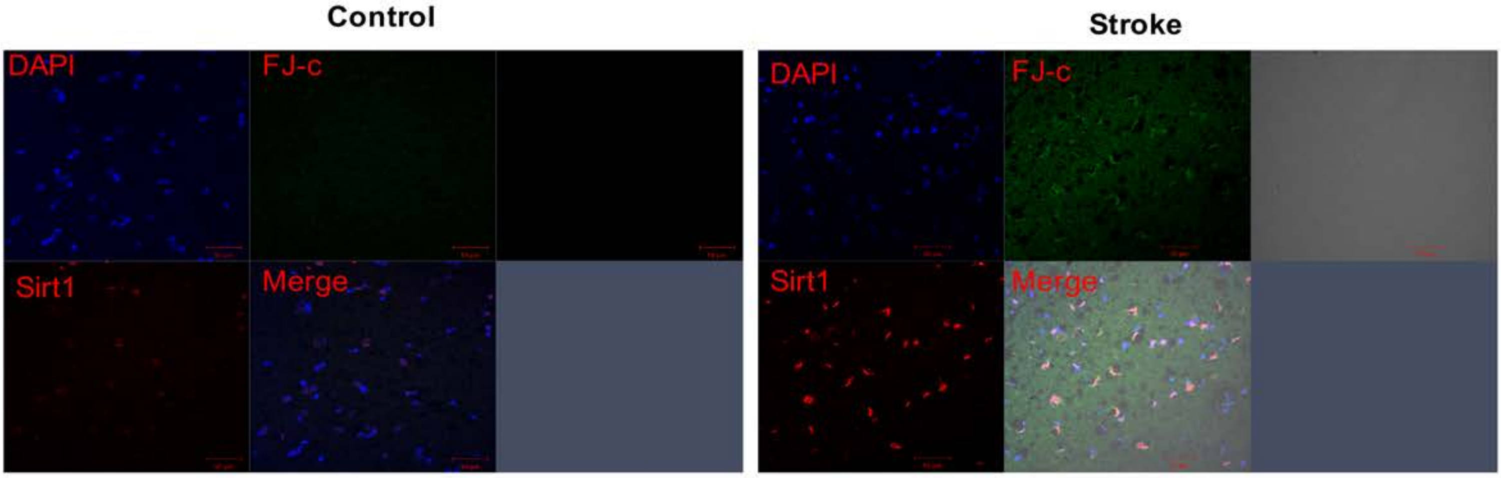

Figure-6: IF in Aged Rats Supplementary

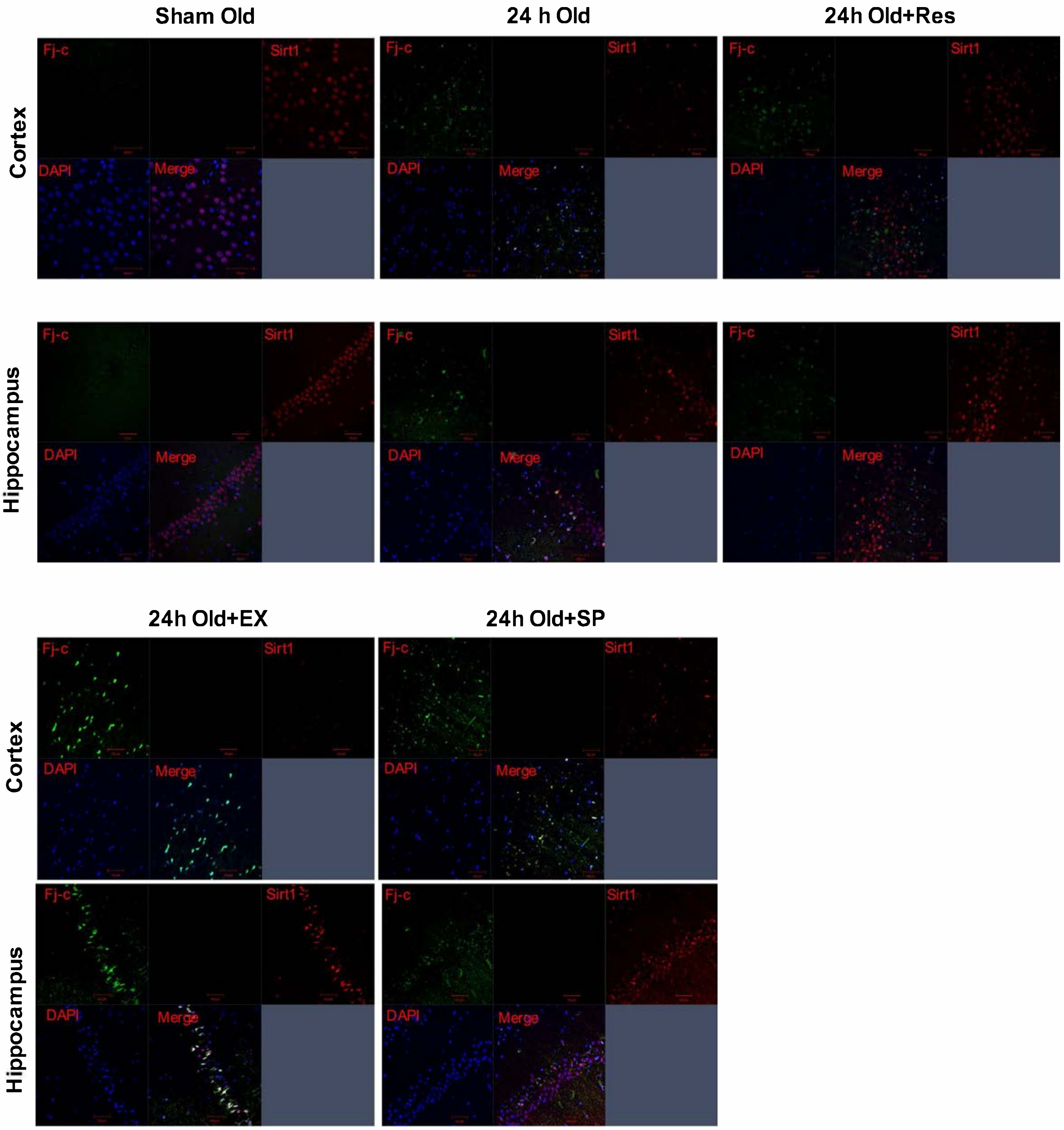

Figure-6 supplementary data: Immuno-fluorescence data quantification for Fluore-Jade-C in aged experimental rats.

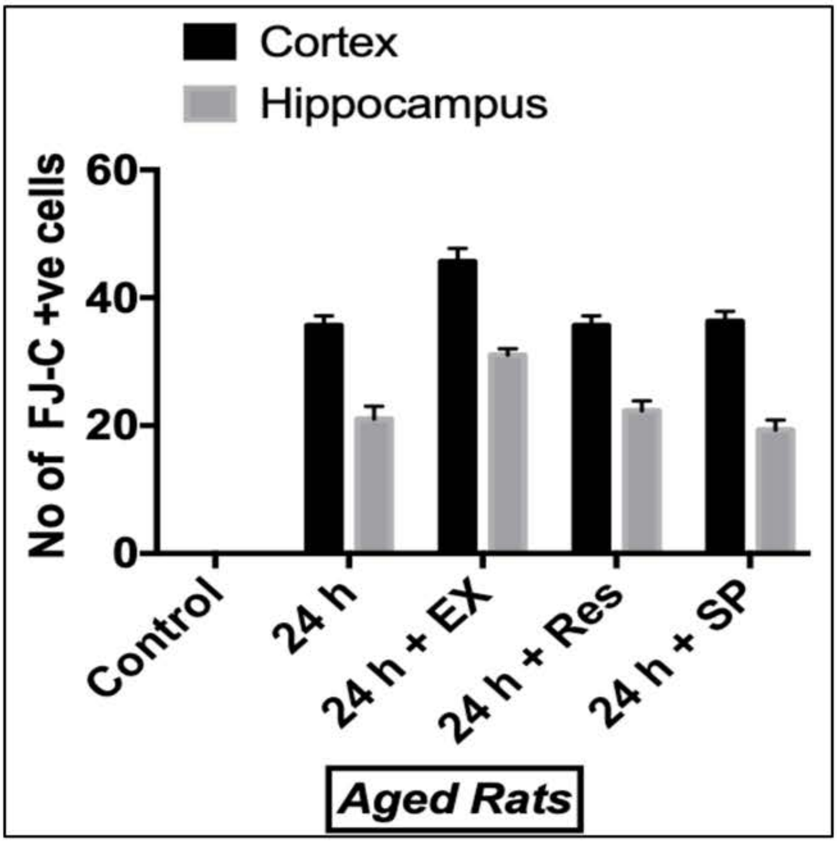

FIgure-7: IF in Young Rats Supplementary

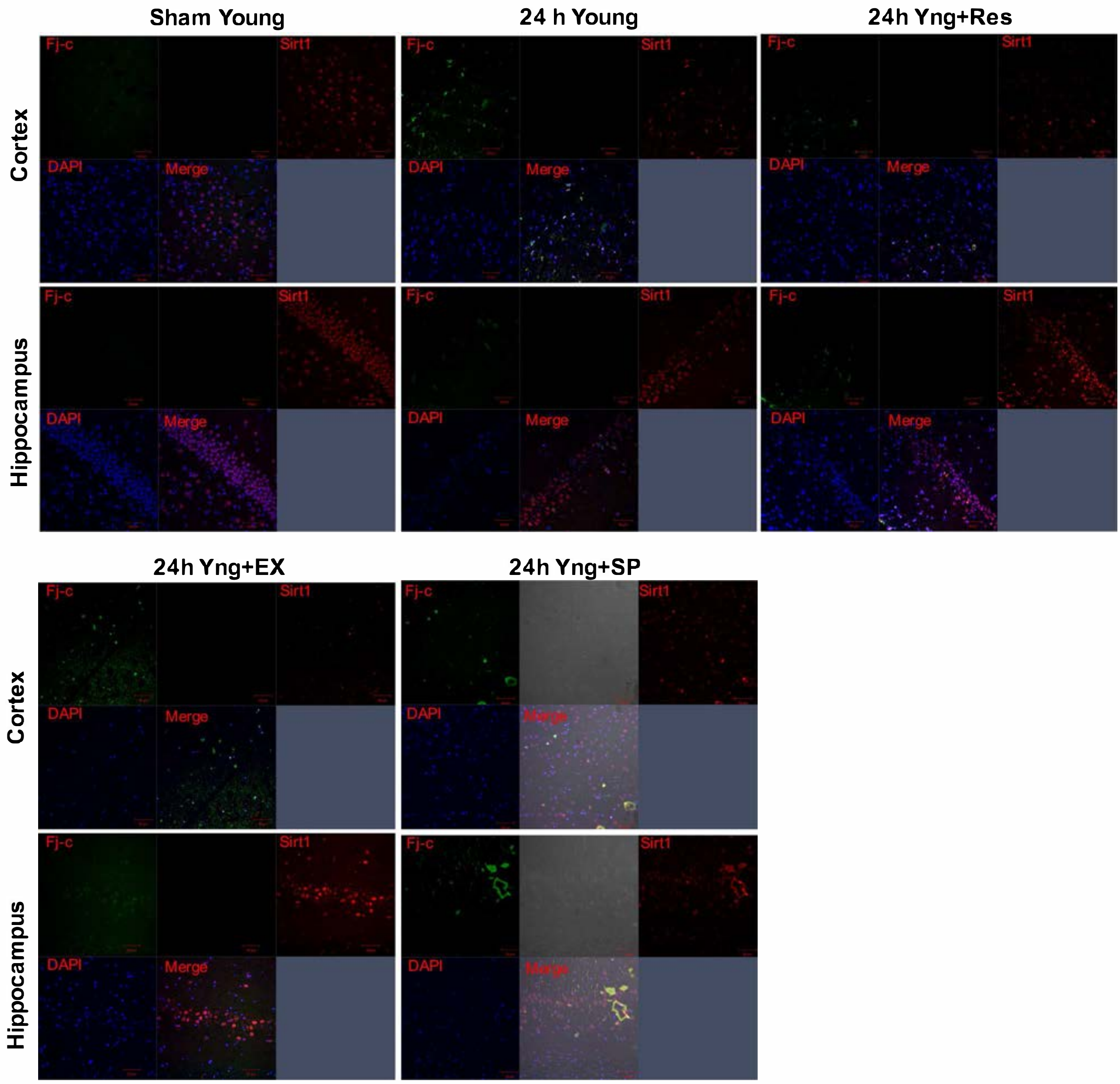

Figure-7 supplementary data: Immuno-fluorescence data quantification for Fluore-Jade-C in young experimental rats.

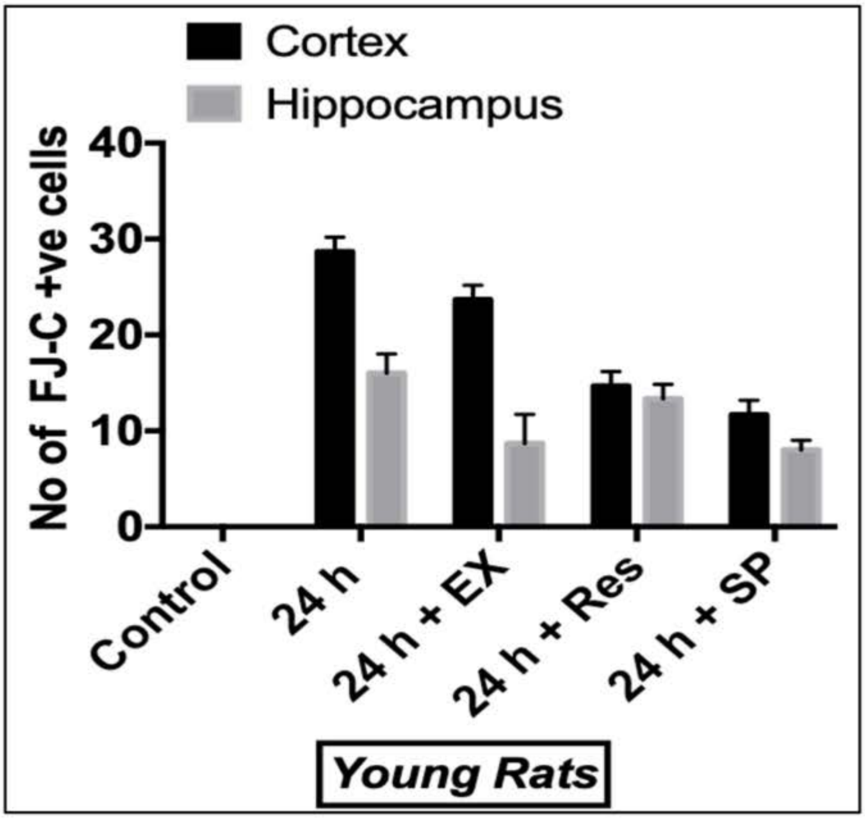

Supplement: Supplementary file 1 — Supplementary figures. [file 41598_2021_85577_MOESM1_ESM.pdf]
